# Brain glucose metabolism in schizophrenia: a systematic review and meta-analysis of ^18^FDG-PET studies in schizophrenia

**DOI:** 10.1017/S003329172200174X

**Published:** 2023-08

**Authors:** Leigh Townsend, Toby Pillinger, Pierluigi Selvaggi, Mattia Veronese, Federico Turkheimer, Oliver Howes

**Affiliations:** 1 Psychiatric Imaging Group, MRC London Institute of Medical Sciences, Hammersmith Hospital, London, UK; 2 Department of Psychosis Studies, Institute of Psychiatry, Psychology & Neuroscience, King's College London, London, UK; 3 Department of Neuroimaging, Institute of Psychiatry, Psychology and Neuroscience, King's College London, London, UK; 4 Azienda Ospedaliero-Universitaria Consorziale Policlinico di Bari, Bari, Italy; 5 Department of Information Engineering, University of Padua, Padua, Italy

**Keywords:** F-18-deoxyglucose (FDG), glucose, metabolism, positron emission tomography (PET), schizophrenia

## Abstract

**Background:**

Impaired brain metabolism may be central to schizophrenia pathophysiology, but the magnitude and consistency of metabolic dysfunction is unknown.

**Methods:**

We searched MEDLINE, PsychINFO and EMBASE between 01/01/1980 and 13/05/2021 for studies comparing regional brain glucose metabolism using ^18^FDG-PET, in schizophrenia/first-episode psychosis *v.* controls. Effect sizes (Hedges *g*) were pooled using a random-effects model. Primary measures were regional absolute and relative CMRGlu in frontal, temporal, parietal and occipital lobes, basal ganglia and thalamus.

**Results:**

Thirty-six studies (1335 subjects) were included. Frontal absolute glucose metabolism (Hedge's *g* = −0.74 ± 0.54, *p* = 0.01; *I*^2^ = 67%) and metabolism relative to whole brain (*g* = −0.44 ± 0.34, *p* = 0.01; *I*^2^ = 55%) were lower in schizophrenia *v.* controls with moderate heterogeneity. Absolute frontal metabolism was lower in chronic (*g* = −1.18 ± 0.73) *v.* first-episode patients (*g* = −0.09 ± 0.88) and controls. Medicated patients showed frontal hypometabolism relative to controls (−1.04 ± 0.26) while metabolism in drug-free patients did not differ significantly from controls. There were no differences in parietal, temporal or occipital lobe or thalamic metabolism in schizophrenia *v.* controls. Excluding outliers, absolute basal ganglia metabolism was lower in schizophrenia *v.* controls (−0.25 ± 0.24, *p* = 0.049; *I*^2^ = 5%). Studies identified reporting voxel-based morphometry measures of absolute ^18^FDG uptake (eight studies) were also analysed using signed differential mapping analysis, finding lower ^18^FDG uptake in the left anterior cingulate gyrus (*Z* = −4.143; *p* = 0.007) and the left inferior orbital frontal gyrus (*Z* = −4.239; *p* = 0.02) in schizophrenia.

**Conclusions:**

We report evidence for hypometabolism with large effect sizes in the frontal cortex in schizophrenia without consistent evidence for alterations in other brain regions. Our findings support the hypothesis of hypofrontality in schizophrenia.

## Introduction

Current drug treatments for schizophrenia predominantly target positive symptoms but are ineffective for many patients and have little effect on negative and cognitive symptoms (Demjaha et al., 2017; Kaar, Natesan, McCutcheon, & Howes, 2020; McCutcheon, Marques, & Howes, 2020). There is a need to understand the pathophysiology underlying symptoms to develop treatments to address them (McCutcheon et al., 2020). Impaired brain metabolism, particularly in the frontal cortex, may be a key component of this pathophysiology. In healthy individuals, there is higher resting metabolism in anterior compared to posterior brain regions (Ingvar & Franzen, 1974; Matsuda et al., 1984). However, in schizophrenia, it is proposed that there is lower metabolism in anterior brain regions compared to posterior regions (Chua & McKenna, 1995; Ingvar & Franzen, 1974). This hypofrontality is thought to predominantly underlie the negative and cognitive symptoms of schizophrenia, partly because of the commonality between these symptoms and the dysexecutive syndrome observed following frontal lobe injury (Frith, 1992; Mortimer, 2005; Mubarik & Tohid, 2016). While multiple individual ^18^FDG-PET studies report that brain glucose metabolism is abnormal in schizophrenia, not all studies of schizophrenia patients have observed that frontal metabolism is decreased (Ben-Shachar et al., 2007; Gur et al., 1995a; Manoach et al., 1999; Soyka, Koch, Möller, Rüther, & Tatsch, 2005).

A meta-analysis by Hill et al. (2004) evaluated evidence for hypofrontality. They found medium effect size reductions in measures of resting and task-activated regional blood flow and metabolism in schizophrenia compared to controls. However, Hill et al. did not separate ^18^FDG-PET studies, measuring glucose metabolism, from other imaging modalities such as Oxygen-15 PET, measuring cerebral blood flow. Thus, it remains unclear if there is lower frontal glucose metabolism in schizophrenia. Furthermore, there is evidence that abnormalities in regional glucose metabolism in schizophrenia are not limited to the frontal cortex. Metabolic abnormalities have been reported in the temporal, parietal, occipital cortices, basal ganglia and thalamus, though the direction of effect is inconsistent (Gur et al., 1987b; Hazlett et al., 1999; Tamminga et al., 1992). Hill et al. did not examine regions outside of the frontal cortex, so it remains unclear whether metabolic changes are seen in other brain regions.

In view of this, we aimed to synthesise the evidence from ^18^FDG-PET studies for abnormal metabolism in schizophrenia. The rate of glucose utilisation can be fully quantified using ^18^FDG-PET imaging as the cerebral metabolic rate of glucose use (CMRGlu) (Phelps et al., 1979; Reivich et al., 1979). Though quantitative ^18^FDG-PET imaging is currently the most accurate *in vivo* method for investigating regional human brain metabolism (Verger & Guedj, 2018), the methodology is complex, therefore more straightforward methods using a simple static acquisition, which produce semi-quantitative measures such as standardised uptake values or uptake ratio, are common (Hamberg et al., 1994). Unfortunately, these measures are weak proxies of brain glucose utilisation, affected by both physiological (e.g. local blood flow variation) and technical variables (Boellaard, 2009). Therefore, our work focussed on fully quantitative measures of glucose utilisation, i.e. absolute and normalised CMRGlu. We performed meta-analyses to assess whether quantitative ^18^FDG-PET studies in schizophrenia support the idea of frontal hypometabolism in schizophrenia compared to healthy subjects. We hypothesised that absolute and relative frontal metabolism would be lower in schizophrenia compared to healthy controls. It has also been proposed that duration of illness, exposure to antipsychotic medication and engagement in tasks may each alter regional brain metabolism (Brugger & Howes, 2017). Therefore, we prespecified subgroup analyses based upon disease chronicity, medication status and resting-state *v.* task-based conditions. We performed synthesis of ^18^FDG-PET studies of regional glucose metabolism in schizophrenia in the temporal, parietal, occipital cortices, basal ganglia and thalamus and performed meta-analyses to assess regional hypometabolism outside the frontal lobes. We also performed signed differential mapping (SDM) analysis on studies which reported MNI or Talairach peak coordinates to investigate subregional changes and to include studies which did not report direct regional quantification of CMRGlu.

## Methods

The protocol for this study was pre-registered on the International Prospective Register of Systematic Reviews (PROSPERO) on 22 April 2020. It can be accessed at: https://www.crd.york.ac.uk/PROSPERO/display_record.php?ID=CRD42020172641.

### Information sources and search strategy

A systematic review was performed according to Preferred Reporting Items for Systematic Reviews and Meta-Analyses (PRISMA) guidelines (Moher, Liberati, Tetzlaff, Altman, & PRISMA, 2010). OVID was used to search MEDLINE, PsychINFO and EMBASE databases to identify manuscripts published from 01/01/1980 until 13/05/2021. Search terms are detailed in online Supplementary Material, Section 1.1.

### Study eligibility

#### Study selection and data extraction

For inclusion, studies were required to report human quantitative ^18^FDG-PET imaging data [CMRGlu (μmol/100 g/min or mg/100 g/min)] for an experimental group of subjects with schizophrenia-spectrum disorders and a healthy control group. Studies where mean and standard deviation values could not be derived were not included. Full inclusion and exclusion criteria are detailed in online Supplementary Materials, Section 1.2.

The process from screening to inclusion was conducted by three independent reviewers (LT, TP and PL). Identified manuscripts were screened using title and abstract. Studies passing screening were subject to full-text review and included if they fulfilled the criteria. Where overlapping studies reported on the same brain regions in the same sample, the study with the largest sample size was included. Reviewers performed data extraction separately using a predetermined electronic template. Data parameters selected for extraction are detailed in online Supplementary Materials, Section 1.3. Discrepancies were settled by consensus.

### Data synthesis

Included studies reported values for brain regions or slices (the study ROIs). These values were extracted and grouped into our *a priori* regions of interest (the meta-analysis ROIs: the frontal, parietal, temporal, occipital lobes, basal ganglia and thalamus). Some studies reported more than one study ROI which fell within a meta-analysis ROI. In this case, the values were pooled to provide a single mean value and standard deviation for a given meta-analysis ROI. Further details of methods used to pool study ROI values are included in online Supplementary Material, Sections 1.4–1.7.

Studies reporting absolute or normalised values for a meta-analysis ROI were allocated to separate absolute or normalised analysis cohorts. There are different methods of normalisation which may be utilised. For this meta-analysis, normalised values obtained by dividing the absolute CMRGlu value for the ROI by a global value of brain CMRGlu, such as whole-brain CMRGlu value, the whole-brain grey matter CMRGlu value or the mean of ROIs across the whole brain, were included. Studies using normalisation to a reference region such as the cerebellum or occipital lobe were not included. Different whole-brain normalisation methods were not separated and were analysed within a single regional normalised metabolism cohort.

Studies reporting absolute CMRGlu used either mg/100 g/min or μmol/100 g/min. For analysis, values were converted to μmol/100 g/min. The formula for this conversion is included in online Supplementary Material, Section 1.8. When values were not presented in text or in tables, where possible mean and standard deviation values were obtained using WebPlotDigitiser (Rohatgi, 2012). Studies reporting comparisons between schizophrenia and healthy controls which could not be included in the quantitative analysis due to reported method of quantification of ^18^FDG uptake or insufficient information were included in a qualitative synthesis (online Supplementary Table S2).

### Statistical analysis

We ran separate meta-analyses for each meta-analysis ROI. Studies reporting absolute and normalised CMRGlu were analysed separately. Primary analyses were only performed if ⩾4 studies were included to maintain power to detect effects (Fu et al., 2011). Random-effects meta-analysis was used to compare the standardised mean difference (Hedge's *g*) between studies with the significance threshold at *p* < 0.05. Analyses were conducted using the R statistical programming language with metafor and dmetar packages (Harrer, Cuijpers, Furukawa, & Ebert, 2019; Team, 2020). Effect sizes were calculated as Hedge's *g*. Hedge's *g* and Cohen's *d* are very similar, though Hedge's *g* is considered more robust in cases where smaller sample sizes are used (*n* < 20). This was the case for some of our studies so Hedge's *g* was considered a better choice (Durlak, 2009; Glen, 2022).

#### Further analyses

Meta-regression was performed to assess the effect of patient age and publication year on the observed results. *I*^2^ was calculated to test for heterogeneity. Outliers were identified using the dmetar package in R. Outlying studies were defined as a study for which confidence interval (CI) does not overlap with the CI of the pooled effect. Meta-analyses with significant outliers were re-run with the outlying studies excluded. Influence analyses were conducted using the leave-one-out-method, in which the result of the meta-analysis is recalculated *K*–1 times, each time leaving out one study to assess the effect of each study on effect size and *I*^2^ heterogeneity (Vietchbauer & Cheung, 2010). Where a significant main effect occurred, subgroup analyses were conducted to explore factors that may contribute to heterogeneity. Mixed-effects model subgroup analyses were performed on cohorts with ⩾4 studies in total with studies divided by disease chronicity, medication status and PET scan condition (task-based or resting state). Subgroup analysis used a ‘*Q* test’ which is an omnibus test. It tests the null hypothesis that all subgroup effect sizes are equal, and is significant when at least two subgroups, or combinations thereof, differ. In cases where omnibus testing was significant, head-to-head (pairwise) subgroup differences were tested statistically using *Z* test following the method detailed in Borenstein, Hedges, Higgins, and Rothstein (2011). Funnel plots were produced for qualitative assessment of publication bias. Egger's regression test (Egger, Davey Smith, Schneider, & Minder, 1997), a commonly used quantitative method that tests for asymmetry in the funnel plot, was used to detect small-study effects that may have biased our results.

### Signed differential mapping analysis

#### Study selection

Studies identified by the search procedure detailed in section ‘Information sources and search strategy’ were screened and those reporting FDG-PET whole-brain voxel-based findings in schizophrenia and/or first-episode psychosis *v.* controls were considered for inclusion. Only studies reporting absolute (as opposed to relative/normalised) ^18^FDG uptake were included in this analysis.

#### Data synthesis

The following information was extracted from considered studies: sample size, whole-brain/regional thresholding values, MNI or Talairach coordinates for peaks; *t*, *z* and/or *p* values for each peak; voxel number for each peak. If this information was not available, the study was excluded from the analysis. Where coordinates were reported as Talairach coordinates, these were converted to MNI space using GingerALE software (https://brainmap.org/ale/) for MacOS using Brett Talairach to MNI conversion. Where *t* values were not reported, *z* and *p* values were converted to *t* values for the purposes of the analysis using the online SDM project statistics converter (https://www.sdmproject.com/utilities/?show=Statistics). Data synthesis followed recommendations made in the paper published by Albajes-Eizagirre et al. (2019).

#### Statistical analysis of signed differential mapping

SDM meta-analysis was conducted using Seed-based d Mapping with Permutation of Subject Images (SDM-PSI) software for MacOS (downloaded from https://www.sdmproject.com/software/) conducted in line with recommendations made in a paper published by Albajes-Eizagirre et al. (2019). Family-wise error correction threshold was set at *p* = 0.05. Results are detailed in section ‘SDM analysis’.

## Results

### Study selection

**Fig. 1.**
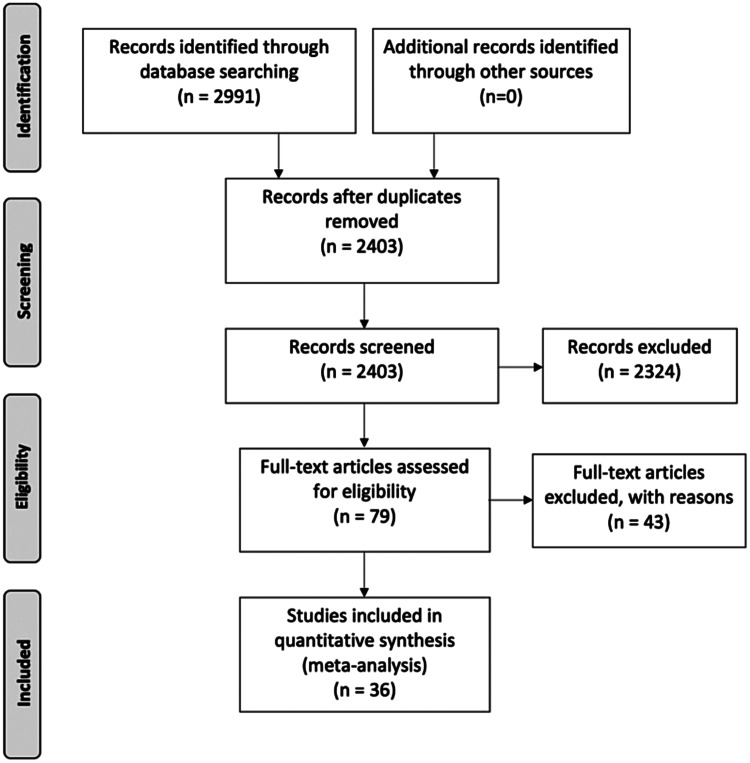
Preferred Reporting Items for Systematic Reviews and Meta-Analyses (PRISMA) flowchart showing study selection process.

The study selection procedure is summarised in Fig. 1. Thirty-six studies (Al-Mousawi et al., 1996; Bartlett et al., 1991; Bertollo, Cowen, & Levy, 1996; Biver et al., 1995; Buchsbaum et al., 1984, 1990, 1992, 1996; Clark, Kopala, Hurwitz, & Li, 1991; Clark, Kopala, Li, & Hurwitz, 2001; Cleghorn et al., 1989; Cohen et al., 1997; Cohen, Nordahl, Semple, Andreason, & Pickar, 1998; Cohen, Nordahl, Semple, & Pickar, 1999; DeLisi et al., 1989; Farkas et al., 1984; Fujimoto et al., 2007; Gur et al., 1995b, 1987a; Hazlett et al., 1998, 1999, 2000; Huret et al., 1991; Jacobsen et al., 1997; Lehrer et al., 2005; Mitelman et al., 2020; Molina, Sanz, Sarramea, Benito, & Palomo, 2005; Nordahl et al., 2001, 1996; Resnick, Gur, Alavi, Gur, & Reivich, 1988; Shihabuddin et al., 1998, 2001; Siegel et al., 1993; Tamminga et al., 1992; Wolkin et al., 1988, 1985), reporting results for 642 patients and 693 healthy controls, were included in the quantitative analysis. Study characteristics are reported in Table 1.

**Table 1.** Table giving details of full text articles included in quantitative analysis

Authors pub. year	Frontal	Parietal	Temporal	Occipital	Basal ganglia	Thalamus	Absolute *v.* normalised – CMRGlu units^a^/normalisation method^b^	Imaging modality	Chronicity of illness – diagnosis	Medication status – minimum duration if drug-free^c^	Task *v.* rest	Type of task – difference patients *v.* controls	Sample size patients/controls	Age matched	Sex distribution % male patients/controls
Buchsbaum et al. (1984)	↑^d^	↔	↔	↔	Not reported	Not reported	Absolute – μmol/100 g/min	PET only	Chronic – schizo-phrenia	Drug free – 14 days	Task	Electrical somato-sensory stimulation – score n/a	16/19	Yes	68%/68%
Farkas et al. (1984)	Not reported	Not reported	Not reported	Not reported	↔	Not reported	Absolute – mg/100 g/min	PET/CT	Chronic – schizo-phrenia	Mixed	Rest	Not applicable	13/11	Yes	100%/100%
Wolkin et al. (1985)	↓	Not reported	Not reported	↓	↔	Not reported	Absolute – μmol/100 g/min	PET only	Chronic – schizo-phrenia	Drug free – 30 days	Rest	Not applicable	10/8	No	100%/100%
Gur et al. (1987a, 1987b)	↓	↓	↓	↓	Not reported	Not reported	Absolute – mg/100 g/min	PET only	Chronic – schizo-phrenia	Naïve/drug free – 6 months	Rest	Not applicable	12/12	Yes	92%/92%
Resnick et al. (1988)	Not reported	Not reported	Not reported	Not reported	↔	↔	Absolute – mg/100 g/min	PET only	Chronic – schizo-phrenia	Mixed	Rest	Not applicable	20/19	Yes	95%/95%
Wolkin et al. (1988)	↓	Not reported	Not reported	↓	Not reported	Not reported	Absolute – μmol/100 g/min	PET only	Chronic – schizo-phrenia	Drug free	Rest	Not applicable	13/8	Yes	100%/100%
Cleghorn et al. (1989)	↔	↔	Not reported	Not reported	Not reported	Not reported	Absolute – μmol/100 g/min	PET only	First-episode psychosis	Naïve	Rest	Not applicable	8/10	Yes	75%/100%
DeLisi et al. (1989)	Not reported	Not reported	↑	Not reported	Not reported	Not reported	Absolute – μmol/100 g/min	PET only	Chronic – schizo-phrenia	Drug free – 15 days	Task	Electrical somato-sensory stimulation – score n/a	21/9	Yes	71%/33%
Buchsbaum et al. (1990)	↔	Not reported	Not reported	↔	Not reported	Not reported	Absolute – μmol/100 g/min	PET only	Chronic – schizophrenia	Drug free – 30 days	Task	Continuous performance task – no sig. difference	13/18	Yes	85%/77%
Bartlett et al. (1991)	↓	↓	↓	↓	↔	↔	Absolute – μmol/100 g/min	PET only	Chronic – schizophrenia	Drug free – duration not spec.	Rest	Not applicable	8/11	No	100%/100%
Huret et al. (1991)	↓	↓	↓	↓	Not reported	Not reported	Absolute – mg/100 g/min	PET only	Chronic – schizophrenia	Medicated	Rest	Not applicable	12/6	Yes	66%/50%
Clark et al. (1991)	↓	↓	↔	Not reported	↔	↔	Absolute – mg/100 g/min	PET only	Chronic – schizophrenia	Medicated	Rest	Not applicable	16/8	Yes	100%/100%
Buchsbaum et al. (1992)	↔	↔	Not reported	↔	↔	Not reported	Absolute – μmol/100 g/min	PET only	First-episode psychosis	Naïve	Task	Continuous performance task – signal detection measure – Pts worse than controls Pts = 2.11 ± 1.3 Ctrls = 2.8 ± 0.8 *p* = 0.034	18/20	Yes	100%/100%
Tamminga et al. (1992)	↔	↔	↔	↔	↔	↔	Absolute – mg/100 g/min	PET only	Chronic – schizophrenia	Drug free	Rest	Not applicable	12/12	Yes	66%/66%
Siegel et al. (1993)	Not reported	↑	↑	Not reported	↑	↑	Absolute – μmol/100 g/min	PET only	Chronic – schizophrenia	Drug free – 4 weeks	Task	Continuous performance task – no sig. difference	70/30	Yes	100%/100%
Biver et al. (1995)	↔	↔	↔	↔	↔	↔	Absolute – μmol/100 g/min	PET only	Chronic – schizophrenia	Naïve/drug free – 6 months	Rest	Not applicable	15/15	Yes	66%/60%
Gur et al. (1995a, 1995b)	↔	↔	↔	↔	↔	↔	Absolute – mg/100 g/min	PET only	Mixed	Mixed	Rest	Not applicable	22/42	Yes	69%/69%
Bertollo et al. (1996)	↓	Not reported	Not reported	Not reported	Not reported	Not reported	Absolute – mg/100 g/min	PET only	Chronic – schizophrenia	Mixed	Rest	Not applicable	8/8	Yes	100%/100%
Al-Mousawi et al. (1996)	↔	Not reported	↔	↑	↓	↔	Normalised – by area weighted mean of all ROIs	PET/MRI	Mixed	Drug free	Rest	Not applicable	13/18	Yes	84%/77%
Buchsbaum et al. (1996)	Not reported	Not reported	Not reported	Not reported	Not reported	↔	Absolute – μmol/100 g/min	PET/MRI	First-episode psychosis	Naïve	Task	Continuous performance task – no sig. difference	20/15	Yes	95%/80%
Nordahl et al. (1996)	↔	Not reported	↔	Not reported	↓	Not reported	Normalised – by whole slice cortical grey matter CMRGlu	PET only	Chronic – schizophrenia	Drug free – 14 days	Task	Stroop – no sig. difference	18/11	Yes	94%/91%
Cohen et al. (1997)	↓	↑	↑	Not reported	Not reported	Not reported	Normalised – by whole slice grey matter CMRGlu	PET only	Chronic – schizophrenia	Medicated	Task	Continuous performance task – score not given	33/41	Yes	100%/100%
Jacobsen et al. (1997)	↔	↔	↔	↔	↔	↔	Normalised by whole-brain grey matter CMRGlu	PET/MRI	Chronic – schizophrenia	Drug free – not stated	Task	Continuous performance task – hit rate – Pts worse than controls Pts = 83.5 ± 63.0 Ctrls = 139.6 ± 40.7 *p* < 0.01	16/26	Yes	62%/42%
Cohen et al. (1998)	Not reported	Not reported	Not reported	Not reported	↑	Not reported	Normalised – by whole-brain grey matter CMRGlu	PET only	Chronic – schizophrenia	Medicated	Task	Continuous performance task – Pts worse than controls values not given	19/41	Yes	66%/62%
Hazlett et al. (1998)	↓	↔	↔	↔	Not reported	Not reported	Normalised – by whole-brain activity	PET only	Mixed	Naïve/drug free – 14 days	Task	Startle eyeblink modification – no sig. difference	16/15	Yes	68%/60%
Shihabuddin et al. (1998)	Not reported	Not reported	Not reported	Not reported	↔	Not reported	Normalised – by whole-brain CMRGlu	PET/MRI	Chronic – schizophrenia	Naïve/drug free – 21 days	Task	California verbal learning task – word recall – Pts worse than controls Pts = 7.41 ± 2.94 Ctrls = 13.0 ± 1.84 *p* < 0.05	18/24	Yes	66/62%
Cohen et al. (1999)	↓	↑	↑	Not reported	↑	Not reported	Normalised – by whole slice grey matter CMRGlu	PET only	Chronic – schizophrenia	Medicated	Task	Auditory discrimination task – scores not given	13/40	No	0%/0%
Hazlett et al. (1999)	Not reported	Not reported	Not reported	Not reported	Not reported	↔	Normalised – by whole-brain CMRGlu	PET/MRI	Chronic – schizophrenia	Mixed	Task	Serial verbal learning task – no sig. difference	27/32	Yes	70%/78%
Hazlett et al. (2000)	↔	↔	↔	↔	Not reported	Not reported	Normalised – by whole-brain grey matter CMRGlu	PET/MRI	Mixed	Mixed	Task	Serial verbal learning task – no sig. difference	20/32	Yes	60%/78%
Clark et al. (2001)	↔	↔	↔	↔	↔	↓	Absolute – mg/100 g/min	PET	First-episode psychosis	Naive	Rest	Not applicable	26/32	Yes	76%/37.5%
Nordahl et al. (2001)	↔	Not reported	Not reported	Not reported	Not reported	Not reported	Normalised – by area weighted mean of all ROIs	PET/MRI	Chronic – schizophrenia	Drug free – 14 days	Task	Stroop – Stroop interference – no sig. difference error rate – Pts worse than controls Pts = 16% Ctrls = 6% *p* = 0.02	9/10	Yes	77%/80%
Shihabuddin et al. (2001)	Not reported	Not reported	Not reported	Not reported	↓	Not reported	Normalised – by whole-brain CMRGlu	PET/MRI	Chronic – schizophrenia	Drug free	Task	Serial verbal learning task – score not given	18/24	Yes	66%/63%
Lehrer et al. (2005)	Not reported	Not reported	Not reported	Not reported	Not reported	↔	Normalised – by whole-brain CMRGlu	PET/MRI	Chronic – schizophrenia	Naive	Task	Visual attention task – no sig. difference	12/13	Yes	58%/61%
Molina et al. (2005)	↔	Not reported	Not reported	Not reported	Not reported	Not reported	Normalised – by whole-brain CMRGlu	PET/MRI	First-episode psychosis	Naive	Task	Continuous performance task – score not given	11/16	Yes	62%/53%
Fujimoto et al. (2007)	↓	↔	↔	↔	↑	↔	Normalised – by whole-brain CMRGlu	PET/MRI	Mixed	Medicated	Rest	Not applicable	22/19	Yes	64%/53%
Mitelman et al. (2020)	↔	Not reported	Not reported	Not reported	Not reported	↓	Normalised – by whole-brain CMRGlu	PET/MRI	Mixed	Naïve/drug free – 30 days	Task	Serial verbal learning task – score not given	24/18	Yes	72%/63%

a CMRGlu units given if absolute metabolism reported.

b Normalisation method given if normalised metabolism reported.

c If results for drug-free patients reported, minimum drug-free period is given.

d Arrows refer to effect reported by article for regions.

↑ = significantly higher CMRGlu in schizophrenia.

↔ = no significant difference in CMRGlu.

↓ = significantly lower CMRGlu in schizophrenia.

Sufficient studies were identified to conduct meta-analyses for the frontal, temporal, parietal, occipital lobes, basal ganglia and thalamic metabolism, using reported absolute and normalised CMRGlu measured in μmol/100 g/min. Studies excluded from the quantitative analysis, including studies which reported regional values for which there were insufficient study numbers to allow meta-analysis, are listed in online Supplementary Table S1. A summary of the number of excluded studies reporting findings for each region and the direction of effect reported is shown in online Supplementary Table S2.

### Frontal lobe metabolism

#### Absolute metabolism

**Fig. 2.**
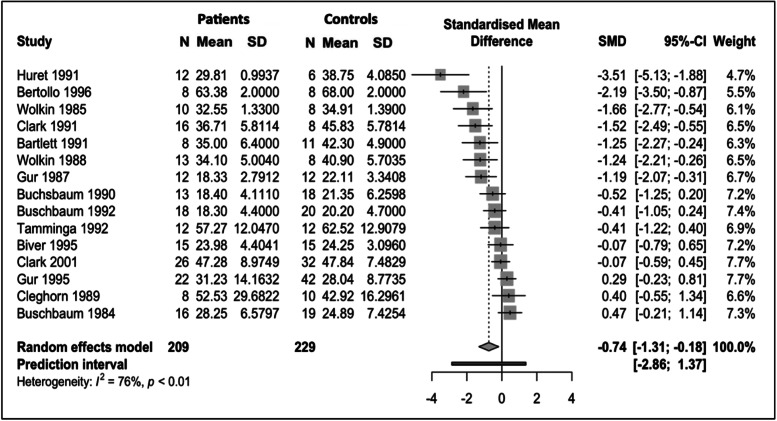
Meta-analysis of studies reporting frontal lobe absolute cerebral metabolic rate for glucose (CMRGlu). Forest plot showing significantly lower CMRGlu in schizophrenia with a large effect size (*g* = −0.74).

Data for absolute glucose metabolism in the frontal lobes from 15 studies (229 controls, 209 patients) were included. Pooled results demonstrate statistically significant lower absolute metabolism in the frontal lobes in schizophrenia relative to controls (SMD = −0.74; 95% CI −1.31 to −0.18; *p* = 0.01) (Fig. 2). *I*^2^ was 76%.

Influence analysis (online Supplementary Fig. S1) showed that no study was exerting a disproportionate effect on the pooled SMD and the upper boundary of 95% CIs remained below zero for pooled SMD for each omission. Two studies (Gur et al. 1995a, 1995b; Huret et al., 1991) were found to contribute most to *I*^2^ heterogeneity. Huret et al. (1991) was also found to be an outlier as the study's CI did not overlap with the CI of the pooled effect. A sensitivity analysis was conducted (online Supplementary Fig. S3) excluding these two studies. Pooled SMD continued to support a statistically significant decrease in absolute metabolism in patients relative to controls (SMD = −0.66, 95% CI −1.14 to −0.18; *p* = 0.01) despite removal of two studies. *I*^2^ heterogeneity was reduced to 67%.

#### Normalised metabolism

**Fig. 3.**
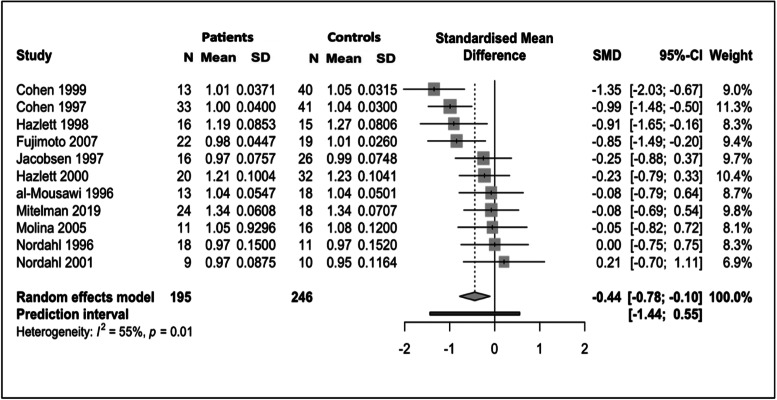
Meta-analysis of studies reporting frontal lobe normalised cerebral metabolic rate for glucose (CMRGlu). Forest plot showing significantly lower CMRGlu in schizophrenia with a moderate effect size (*g* = −0.44).

Data for normalised glucose metabolism in the frontal lobes from 11 studies (246 controls, 195 patients) were included. Pooled results showed a statistically significant decrease in normalised frontal glucose metabolism (frontal cortex relative to whole-brain glucose metabolism) in patients with schizophrenia relative to controls (SMD = −0.44; 95% CI −0.78 to −0.10; *p* = 0.01) (Fig. 3). *I*^2^ was 55%.

No outliers were identified and influence analysis (online Supplementary Fig. S2) showed that no study was exerting a disproportionate effect on the pooled SMD. The upper boundary of 95% CIs remained below zero for pooled SMD for each omission.

#### Subgroup analyses and meta-regression

Detailed results for subgroup analyses for the frontal region can be found in online Supplementary Material, Section 2.

##### Disease chronicity

For subgroup analysis based on disease chronicity (online Supplementary Material, Section 2.1), cohorts were divided into three subgroups – chronic patients, first-episode patients and mixed. For absolute frontal metabolism, the test for subgroup differences (online Supplementary Fig. S4.1) showed a significant difference between subgroups [*Q* = 11.58, degrees of freedom (df) = 2, *p* < 0.01]. Pairwise comparison of SMD for absolute chronic studies (11 studies) *v.* absolute First Episode Psychosis (FEP) studies (three studies) using *Z* test did not find a significant difference between groups (*Z* = 0.89, *p* = 0.38). Comparison with absolute mixed subgroup was not conducted due to small study sample number.

For normalised metabolism (online Supplementary Fig. S5.1), there was not a significant difference between subgroups (*Q* = 0.96, df = 2, *p* = 0.62).

##### Task *v.* rest

For subgroup analysis between task-based and resting-state conditions (online Supplementary Material, Section 2.2), a significant difference between subgroups was not observed for the absolute frontal metabolism (online Supplementary Fig. S4.2) or normalised (online Supplementary Fig. S5.2) cohorts.

##### Medication status

For subgroup analysis based on medication status (online Supplementary Material, Section 2.3), cohorts were divided into five subgroups: drug free, medicated, mixed (containing medicated and drug-free patients), drug naïve and naïve/drug free. The test for subgroup differences in the frontal absolute cohort did not show a significant difference between groups (*Q* = 7.39, df = 4, *p* = 0.12). In the normalised cohort (online Supplementary Fig. S5.3), the test for subgroup differences showed a significant difference between groups (*Q* = 36.46, df = 4, *p* < 0.01). Due to small numbers of studies available for mixed, drug-naïve and naïve/drug-free groups, pairwise comparison for subgroups was only conducted for normalised drug-free studies (four studies) *v.* normalised medicated studies (three studies) using *Z* test. This found a significant difference between normalised SMD for drug-free *v.* medicated studies (*Z* = 3.08, *p* < 0.01), with lower normalised frontal glucose uptake in studies of patients taking antipsychotic medication relative to those in antipsychotic-free cohorts.

A further analysis was performed pooling the subgroups into two larger subgroups: medicated/mixed and naïve/drug free for absolute (online Supplementary Fig. S4.4) and normalised cohorts (online Supplementary Fig. S5.4). In the absolute metabolism cohort, no significant difference was observed between subgroups (*Q* = 1.89, df = 1, *p* = 0.16). In the normalised metabolism cohort, there was a significant difference between the two groups (*Q* = 6.2, df = 1, *p* = 0.01). Pairwise comparison of SMD for normalised medicated/mixed studies (four studies) *v.* normalised naïve/drug-free studies (seven studies) using *Z* test did not find a significant difference between the groups (*Z* = 1.64, *p* = 0.1).

##### Meta-regression

Meta-regression analyses were conducted to assess the effect of patient age and study publication year on pooled SMD. For the frontal absolute metabolism cohort, patient age (*p* = 0.35) and publication year (*p* = 0.72) were not significant moderators of effect. Similarly, for the frontal normalised metabolism cohort, patient age (*p* = 0.17) and publication year (*p* = 0.49) were not significant moderators of effect.

##### Funnel plots and Egger's test

Funnel plots were produced for frontal absolute (with outliers removed) (online Supplementary Fig. S5.5) and frontal normalised cohorts (online Supplementary Fig. S5.6). Visual inspection did not identify significant deviations from the funnel shape. For quantitative analysis of small-study effects, Egger's test was used. For the frontal absolute cohort (with outliers removed), Egger's test showed an intercept of −5.12 (CI −7.87 to −2.38, *t* = −3.64, *p* < 0.01) suggesting that there was a significant small-study effect on pooled SMD in this cohort.

For the frontal normalised cohort, Egger's test showed an intercept of 3.62 (CI −1.67 to 8.92, *t* = 1.36, *p* = 0.21) suggesting that there was no significant small-study effect on pooled SMD in this cohort.

### Metabolism in other regions

We did not find significant differences in metabolism in most other brain regions (see Table 2). A full description of results for absolute and normalised analyses for the temporal, parietal, occipital lobes and thalamus can be found in online Supplementary Material, Section 3. A significant difference in basal ganglia metabolism between patients and controls was identified following the exclusion of an outlier. Results for basal ganglia metabolism are detailed below.

**Table 2.** Table summarising findings for non-frontal regional meta-analyses

ROI	Absolute *v.* normalised	Studies (*n*)	Controls (*n*)	Patients (*n*)	Hedge's *g* (SMD)	95% Confidence interval (for SMD)	*p* value (for SMD)	Direction of effect (relative to controls)	With outliers removed (effect relative to controls)
Temporal lobe	Absolute	11	206	230	−0.23	[−0.75; 0.30]	0.36	↔	No outliers
	Normalised	8	202	151	0.35	[−0.03; 0.74]	0.07	↔	No outliers
Parietal lobe	Absolute	12	217	255	−0.45	[−1.10; 0.20]	0.16	↔	↔ SMD = −0.33; 95% CI [−0.76; 0.11] *p* = 0.13
	Normalised	6	173	109	0.17	[−0.45; 0.80]	0.51	↔	No outliers
Occipital lobe	Absolute	12	203	207	−0.55	[−1.12; 0.01]	0.06	↔	No outliers
	Normalised	5	92	74	0.20	[−0.48; 0.89]	0.46	↔	No outliers
Basal ganglia	Absolute	11	201	251	−0.13	[−0.47; 0.21]	0.97	↔	↓ SMD = −0.25; 95% CI [−0.49, −0.0014] *p* = 0.049*
	Normalised	8	211	146	−1.04	[−4.35; 2.26]	0.48	↔	↔ SMD = 0.22; 95% CI [−0.81, 1.25] *p* = 0.39
Thalamus	Absolute	9	176	229	−0.08	[−0.48; 0.32]	0.66	↔	↔ SMD = −0.22; 95% CI [−0.51; 0.06] *p* = 0.11
	Normalised	6	127	115	−0.34	[−0.90; 0.22]	0.18	↔	no outliers

↑ = significantly higher CMRGlu in schizophrenia.

↔ = no significant difference in CMRGlu.

↓ = significantly lower CMRGlu in schizophrenia.

#### Basal ganglia metabolism

Pooled results for absolute glucose metabolism in the basal ganglia including data from 11 studies (251 controls, 201 patients) did not demonstrate a statistically significant difference in absolute glucose metabolism (SMD = −0.13; 95% CI −0.47 to 0.21; *p* = 0.97, online Supplementary Fig. S6.1) nor did the analysis for normalised glucose metabolism which included eight studies (211 controls, 146 patients) (SMD = −1.04; 95% CI −4.35 to 2.26; *p* = 0.48, online Supplementary Fig. S7). *I*^2^ was 64% (moderate heterogeneity) for the analysis of absolute glucose metabolism and 94% (substantial heterogeneity) for the normalised analysis.

For the absolute cohort, a study by Siegel et al. (1993) was found to be an outlier and significantly contributed to heterogeneity. A further random-effects meta-analysis excluding this study (online Supplementary Fig. S6.2) showed a pooled SMD for absolute basal ganglia metabolism of −0.25 (95% CI −0.49 to −0.0014; *p* = 0.0489). *I*^2^ was reduced to 5% (low heterogeneity). For the normalised cohort, a study by Al-Mousawi et al. (1996) was found to be an outlier; however, a further random-effects meta-analysis excluding this study remained non-significant with a pooled SMD of 0.22 (95% CI −0.81 to 1.25) and heterogeneity remained high (*I*^2^ = 90%).

### SDM analysis

#### Study selection

Of 79 full-text studies assessed for eligibility, 10 studies were identified as eligible for SDM analysis (Choudhary et al., 2015; Desco et al., 2003; Fernandez-Egea et al., 2010; Horacek et al., 2004; Horga et al., 2014; Molina, Sanz, Sarramea, & Palomo, 2007; Park et al., 2009; Potkin et al., 2002; Ben-Shachar et al., 2007; Kim et al., 2017). These studies reported results for 234 patients and 120 controls. Two studies (Ben-Shachar et al., 2007; Kim et al., 2017) considered for inclusion were excluded, as they reported relative rather than absolute FDG uptake.

#### SDM results

**Fig. 4.**
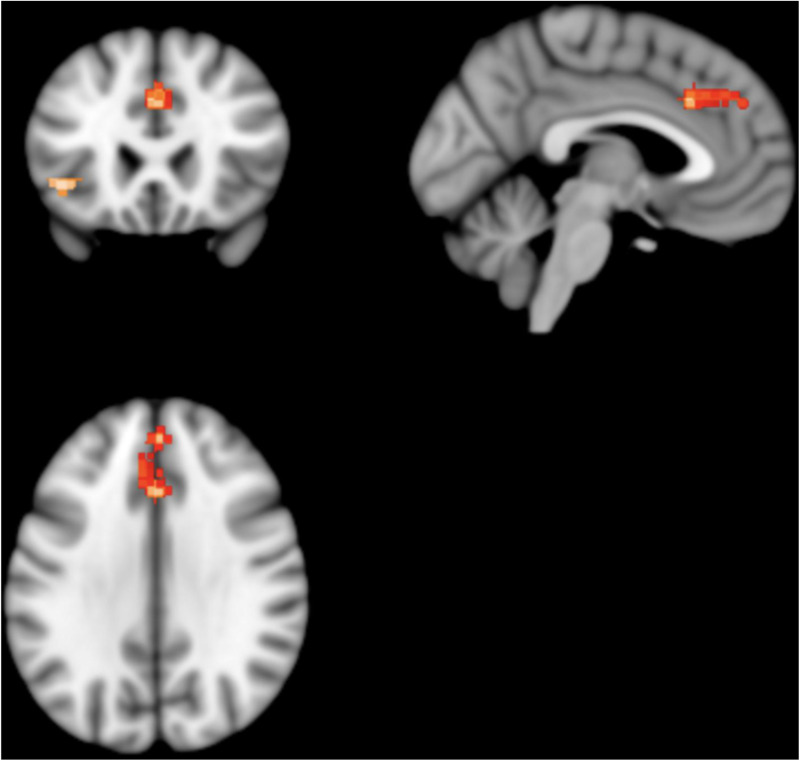
Results from SDM analysis – regions of statistically significantly lower ^18^FDG uptake volume in patients with schizophrenia as compared to matched healthy controls: two clusters of statistical significance are present. The larger cluster contains the left cingulate/paracingulate gyrus and medial left superior frontal gyrus. It consists of 309 voxels and its peak coordinates are at MNI [0, 24, 34], (*Z* = −4.143, *p* = 0.007). The smaller cluster contains the orbital part of the left inferior frontal gyrus. It consists of 62 voxels and its peak coordinates are at MNI [−42, 24, −4], (*Z* = −4.239, *p* = 0.02).

Patients with schizophrenia showed two clusters of statistically significantly lower absolute ^18^FDG uptake; one in the left median cingulate/paracingulate gyri and the other in the left inferior orbital frontal gyrus (Fig. 4).

The left median cingulate/paracingulate gyri cluster consisted of 309 voxels with the cluster peak at MNI [ 0, 24, 34] (*Z* value = −4.1, FWE-corrected *p* value = 0.007). Subclusters consisted of the left medial superior frontal gyrus (161 voxels), left median cingulate/paracingulate (73 voxels), right median cingulate (58 voxels), right superior frontal gyrus (13 voxels) and left anterior cingulate (nine voxels). The left inferior orbital frontal gyrus cluster consisted of 62 voxels with the cluster peak at MNI [−42, 24, −4] (*Z* value = −4.2, FWE-corrected *p* value = 0.02). Subclusters consisted of the left inferior frontal gyrus, orbital part (32 voxels), left inferior frontal gyrus, triangular part (23 voxels) and left insula (seven voxels). No other significant differences were identified.

Assessing for heterogeneity and publication bias, *I*^2^ was 3.7% (low heterogeneity) and the funnel plot did not demonstrate significant asymmetry, indicating low risk of publication bias.

## Discussion

This study finds that absolute and normalised measures of frontal glucose metabolism are lower in schizophrenia, with moderate–large effect sizes (−0.74 for absolute CMRGlu and −0.44 for normalised CMRGlu), relative to controls. This is consistent with the hypothesis of hypofrontality in schizophrenia. Another key finding is that effects were significantly different between subgroups based on medication status, with greater normalised hypometabolism (relative to controls) in medicated patients compared to drug-free patients, who did not differ from controls. We also found that chronic patients showed lower absolute frontal metabolism relative to controls, while first-episode patients did not differ from controls; however, pairwise comparison of chronic *v.* first-episode studies did not find a significant difference between these subgroups. Meanwhile, meta-regression did not identify patient age as a significant moderator of brain glucose metabolism regions. Thus, our findings suggest a potential role of antipsychotic treatment and illness duration in the lower frontal metabolism seen in schizophrenia; however, additional studies comparing first-episode and chronic groups as well as drug-naïve and medicated groups are needed to test this further.

Meta-analysis of fMRI studies using cognitive tasks has found altered frontal cortical activation in schizophrenia relative to controls (Minzenberg, Laird, Thelen, Carter, & Glahn, 2009), and studies in high-risk populations also show frontal abnormalities (Lord et al., 2012, 2011). Our study extends these findings, indicating that lower glucose metabolism could explain these frontal functional abnormalities, and extends other evidence for glucoregulatory abnormalities in schizophrenia (Pillinger et al., 2017). The previous meta-analysis by Hill et al. (2004) pooled results from studies indexing cerebral blood flow and studies assessing regional cerebral glucose metabolism using ^18^FDG-PET, including semi-quantitative measures of ^18^FDG uptake. They reported an effect size (Hedge's *g*) of −0.55 for absolute measures and −0.32 for normalised measures of blood flow/metabolism in schizophrenia relative to controls. Our meta-analysis is the first to focus on quantitative measures of glucose metabolism, and our findings, with effect sizes of −0.74 for absolute and −0.44 for normalised measures of frontal CMRGlu, are larger than the effects reported by Hill et al. (2004), possibly indicating that gluco-metabolic abnormalities may be more marked than abnormalities in blood flow. Notably, however, the Hill et al. meta-analysis did not include more modern methods of measuring regional cerebral blood flow (rCBF), namely arterial spin labelling (ASL). ASL compares favourably with PET measures of rCBF, facilitating quantitative measurement of rCBF without the need for radiation exposure (Fan, Hesamoddin, Holdsworth, & Zaharchuk, 2016) and is increasingly being used to measure rCBF in schizophrenia. While individual studies report frontal hypoperfusion in schizophrenia (Oliveira et al., 2018; Zhu et al., 2015), among other regional abnormalities in blood flow (Guimares, Machado-de-Sousa, Crippa, Guimares, & Hallak, 2016), to our knowledge, a quantitative meta-analysis of ASL studies in schizophrenia has not been published to date. Therefore, it remains to be seen how ASL-measured rCBF compares to our findings of frontal hypometabolism. A recent bimodal meta-analysis (Sukumar, Sabesan, Anazodo, & Palaniyappan, 2020) using SDM to collate results from ASL and FDG-PET voxel-based morphometry studies indicates that discrepancies between blood flow and glucose metabolism, ‘neurovascular uncoupling’, occur in the frontal lobe in schizophrenia. We suggest that dual-scanning studies in the same patients, using fully quantitative measures of glucose metabolism (i.e. CMRGlu) and blood flow (e.g. ASL) would be best placed to address the question of whether gluco-metabolic abnormalities co-occur equally with abnormalities of blood flow, and to compare their degree.

We did not find significant alterations in brain regions outside of the frontal lobe. However, it is possible that our sample of studies for non-frontal regions lacked power to distinguish small effects in these regions. Supporting this, after removing outliers, we observed a small, statistically significant reduction in absolute basal ganglia metabolism in schizophrenia relative to controls. Further, though not meeting the *p* < 0.05 significance threshold, the upper bound CI for absolute occipital metabolism was close to zero (SMD = −0.55; 95% CI −1.12 to 0.01). The same was true, after removing outliers, for absolute thalamic metabolism (SMD = −0.22; 95% CI −0.51 to 0.06). Thus, glucose metabolism may show small reductions in other brain regions in schizophrenia. Nevertheless, our finding that frontal metabolism is lower in schizophrenia after accounting for whole-brain glucose metabolism and that there are no overall significant alterations in glucose metabolism in the parietal, temporal or occipital lobes or in the thalamus indicates that lower glucose metabolism is most marked in the frontal cortex in schizophrenia.

The validity of our findings is supported by the additional analysis conducted using a different meta-analytical methodology, SDM analysis. SDM analysis allowed us to pool results from studies reporting peak coordinates for differences in absolute ^18^FDG uptake, the majority of which could not be included in the main analysis as they did not report quantitative measures of CMRGlu. SDM analysis identified a moderate-sized statistically significant cluster of hypometabolism consisting of peaks within the left anterior cingulate and left medial superior frontal gyrus and a smaller cluster within the orbital part of the left inferior frontal gyrus, while no significant areas of hypo- or hyper-metabolism were identified outside the frontal lobes. This corroborates and complements the findings from our main analysis. Our primary method, random-effects meta-analysis, was applied to fully quantitative measures of brain glucose metabolism and as such gives an indication of the degree of metabolic variation which is directly comparable across studies and regions; however, due to the age of many included studies and the changing reporting and parcellation practices over time, fine-grained spatial resolution was not reliably obtainable. While the presence of hypofrontality is widely cited, large-scale supportive evidence of frontal hypometabolism has been lacking. Therefore, our findings of marked absolute and relative hypometabolism ‘on average’ in the frontal lobes in schizophrenia, alongside more spatially specific findings of absolute hypometabolism in regions known to be important for emotional and social cognition (anterior cingulate) (Chayer & Freedman, 2001; Kerns et al., 2004; Stevens et al., 2011) and response inhibition and attentional control (medial superior frontal gyrus, inferior frontal gyrus) (Chayer & Freedman, 2001; Li et al., 2013; Swick, Ashley, & Turken, 2008), provide support for the hypofrontality hypothesis. They also indicate that gluco-metabolic alterations may be most marked within subregions of the frontal cortex, including the cingulate cortex and inferior frontal gyrus. More systematic characterisation of frontal lobe metabolic function using fully quantitative measures of CMRGlu is needed. While extensive multiple region of interest measurements are not always feasible due to resource constraints, when measuring frontal CMRGlu, future studies should report absolute and normalised CMRGlu for whole frontal lobes, as well as their chosen subregions of interest. Subregions should be identified using standardised neuroimaging atlases and/or should be identified in reporting with standardised coordinates (e.g. MNI coordinates) in order to allow reproduction and comparison of results between studies.

### Strengths and limitations

Our study is the first meta-analysis to specifically investigate glucose metabolism in schizophrenia, addressing both frontal glucose metabolism, which has not been considered alone in previous meta-analyses and glucose metabolism outside the frontal lobe, which has not been assessed by meta-analysis prior to our study. We employed screening criteria to exclude semi-quantitative and non-standardised methods of metabolism measurement, improving the reliability of our findings to inform future work.

We used a random-effects, rather than fixed-effects, model which, though less powerful, is more robust to heterogeneity (Borenstein et al., 2011). Most analyses showed moderate to substantial heterogeneity, not attributable to prominent outliers. Significant subgroup differences for disease chronicity and medication status suggest that systematic differences in observed glucose metabolism in schizophrenia, linked to these factors, may account for some of this heterogeneity. Methodological differences may explain some of the heterogeneity. Older studies tended to parcellate images from two-dimensional slices while later studies tended to define three-dimensional regions of interest, although we did not identify publication year as a significant moderator, suggesting this is not a major factor.

Egger's test for small-study effects showed a statistically significant bias in the frontal absolute cohort (with outliers removed). While this may suggest a bias in publication towards studies finding hypofrontality in schizophrenia, this bias was not identified for the frontal normalised cohort. There are also ‘benign’ causes which may result in similar patterns (Page, Sterne, Higgins, & Egger, 2020). Most notably, asymmetry can be caused by between-study heterogeneity. Funnel plots assume that the dispersion of effect sizes is caused by the studies' sampling error, but do not control for the fact the studies may be estimators of different true effects.

The interpretation of our findings may also be complicated by alterations in grey matter volume which occur in schizophrenia (Brugger & Howes, 2017), and decreased brain volume may have contributed to lower frontal CMRGlu. Only one included study (Fujimoto et al., 2007) used methodology to account for variations in grey matter, and still found lower frontal CMRGlu in schizophrenia with an effect size of −0.85 (95% CI −1.49 to −0.20). Further, the observed effects for lower frontal metabolism we have reported are larger than the decreases observed for brain structural volumes (Brugger & Howes, 2017) and a consistent pattern of CMRGlu reduction is not seen in all regions where brain structural effects have been observed. Thus, brain volumetric differences are unlikely to account for much of the effects we see.

The relationship between regional glucose metabolism and symptom severity, including cognitive or executive dysfunction, was not assessed due to insufficient reporting of these factors in our sample of studies. Though we did not observe a significant difference between task-based and resting-state studies of frontal metabolism; only three included studies reported task-based imaging in the absolute cohort and only three reported resting-state imaging in the normalised cohort. It is likely that our subgroup comparisons were underpowered to detect a difference between task and rest CMRGlu in either cohort. Results may also have been skewed by a higher proportion of resting-state studies reporting results from chronic patients, who we found to show significantly reduced frontal CMRGlu. Similarly, while we observed significant subgroup differences due to medication status in normalised frontal metabolism, illness duration may confound this as patients who were drug naïve were mostly also in their first episode of illness. These factors require further study.

### Interpretation and implications for understanding schizophrenia

^18^FDG is transported into brain cells and begins the gluco-metabolic process in the same way as unlabelled glucose. However, unlike glucose, ^18^FDG cannot be processed further and accumulates in the cells (Dienel, 2019). Thus, the rate of ^18^FDG accumulation directly reflects the rate of glucose uptake and utilisation in a given brain region (Dienel, 2019). A significant proportion of brain glucose utilisation is attributable to the maintenance of the balance between excitatory and inhibitory synaptic activity (Shah et al., 2017; Sohal & Rubenstein, 2019; Tomasi, Wang, & Volkow, 2013). This is reflected by the fact that the ^18^FDG-PET signal is predominantly observed in grey matter structures (Atwell & Laughlin, 2001; Mergenthaler, Lindauer, Dienel, & Meisel, 2013). While ^18^FDG-PET lacks spatial resolution to distinguish between neurons or astrocytes, as 60–80% of the total energy consumption in the brain during resting state is devoted to glutamate cycling (Raichle & Gusnard, 2002; Rajmukar et al., 2021), it is likely that the ^18^FDG-PET signal measures the energy demands for maintenance of neuronal functional integrity. By contrast, energy consumption associated with changes in brain activity, such as task activation is small, less than 5% of baseline (Raichle & Mintun, 2006). As such, our findings of lower absolute and normalised frontal CMRGlu in schizophrenia are indicative of a baseline abnormality in neuronal functioning in the frontal cortex both in absolute terms and relative to the whole brain, while our observation of a smaller effect for normalised frontal CMRGlu compared to absolute frontal CMRGlu suggests that frontal hypometabolism may occur on the background of a more widespread, although less marked, reduction in CMRGlu in the brains of individuals with schizophrenia. A finding from the meta-analysis by Hill et al. corroborates this, reporting a small but significant reduction in whole-brain blood flow/metabolism of −0.26 in schizophrenia (Hill et al., 2004).

One explanation for our findings could be there are specific abnormalities affecting metabolic processes in schizophrenia which particularly affect glucose utilisation in the frontal cortex. Schizophrenia is associated with abnormal mitochondrial function and mitochondrial genetic and structural variation (Zuccoli, Saia-Cereda, Nascimento, & Martins-de-Souza, 2017). A meta-analysis by Whitehurst et al. (Whitehurst et al., 2020) finds that decreases in N-acetyl aspartate (NAA) levels (as measured by ^1^H-MRS), a marker of neuronal metabolic function, are localised to the frontal lobe in early psychotic illness, while reductions in NAA in chronic schizophrenia also affect the temporal lobe, parietal lobe and hippocampus as well as frontal white matter. Another potential explanation is that hypometabolism is secondary to decreased demand due to abnormal synaptic function. Converging evidence implicates synaptic dysfunction in the pathophysiology of schizophrenia, including post-mortem (Osimo, Beck, Marques, & Howes, 2019) and *in vivo* evidence for lower frontal synaptic protein levels (Onwordi et al., 2020). Lower synaptic activity could result in decreased neuronal glucose demand. Studies testing whether glucose metabolic abnormalities are linked to mitochondrial or synaptic dysfunction in schizophrenia are needed to investigate these explanations further.

Although hypofrontality is proposed as explanation for negative and cognitive symptoms in schizophrenia, the relationship between CMRGlu and negative symptoms and cognitive abnormalities attributable to frontal lobe dysfunction was not sufficiently reported, in the studies included in our analysis, to allow us to evaluate this in our meta-analysis. Future studies should therefore aim to test the hypothesis that impaired frontal metabolism underlies these symptoms. It is important to note that the heterogeneity of symptoms which occur within the schizophrenia syndrome may mask group differences in frontal CMRGlu when comparing schizophrenia to controls. Different frontal subregions are known to serve different functions within distinct, distributed cortico-subcortical circuits (Chayer & Freedman, 2001). Thus, variations in participants' symptom phenotype may be due to differences in the anatomical localisation of metabolic abnormality within frontal regions. Studies comparing symptomatology and task performance for patients in both resting and non-resting conditions are needed to better understand the functional consequences of decreased CMRGlu; however, it is important that these studies perform quantitative assessments of metabolism of multiple frontal lobe subregions, to identify correlations between variations in symptoms and variations in regional and subregional metabolic activity between patients.

Our finding that effects are more marked in chronic patients is intriguing, implying that impairments in frontal metabolism progress during the illness course. Some work has shown that antipsychotics can affect glucose regulation (Howes et al., 2004), and reduce regional brain glucose metabolism (Cochran, McKerchar, Morris, & Pratt, 2002; Colangelo et al., 1997; Kim et al., 2013) and therefore could contribute to effects in patients. This is consistent with our findings of frontal hypometabolism in medicated patients but not in drug-free and drug-naïve patients. However, it should be noted that lower frontal glucose metabolism was also found in studies of chronic patients who were antipsychotic free, indicating that antipsychotic treatment is unlikely to fully account for this finding (Gur et al., 1987a, 1987b; Wolkin et al., 1988, 1985). Longitudinal ^18^FDG-PET studies recruiting first-episode patients relative to controls and performing within-subject comparisons, with and without medication, over the course of the illness will be better placed to examine the relationship between medication, disease chronicity and frontal lobe CMRGlu.

## Conclusions

We report hypometabolism in the frontal cortex in schizophrenia with a large effect size and loss of the normal frontal hypermetabolism relative to other brain regions, but no consistent evidence for alterations in other brain regions examined. Our findings support the hypothesis of hypofrontality in schizophrenia and indicate that metabolic abnormalities progress during illness course with antipsychotics playing a role in this change. Frontal hypometabolism may underlie the negative and cognitive symptoms of schizophrenia, therefore glucose metabolism should be considered as a potential target for drug treatment to address these symptoms.

## Supplementary material

For supplementary material accompanying this paper visit https://doi.org/10.1017/S003329172200174X. click here to view supplementary material
