# Supplementary material for: Brain glucose metabolism in schizophrenia: A systematic review and meta-analysis of 18FDG-PET studies in schizophrenia
Source: Psychol Med. Author manuscript; Available in PMC 2023 Sep 5. (PMC10476075; doi:10.1017/S003329172200174X)

Brain glucose metabolism in schizophrenia: A systematic review and meta-analysis of ^18^FDG-PET studies in schizophrenia - **Supplementary material**

1. SUPPLEMENTARY METHODS
   1. Search strategy

The search terms used were as follows: **(1)** exp schizophrenia/ **(2)** exp psychosis/ **(3)** schizophren*.mp. **(4)** exp fluorodeoxyglucose f 18/ **(5)** metaboli*.mp. (**6)** gluco*.mp. **(7)** FDG.mp. **(8)** 18F - FDG.mp. **(9)** exp positron emission tomography/ **(10)** PET.mp. **(11)** 1 or 2 or 3 **(12)** 4 or 5 or 6 or 7 or 8 **(13)** 9 or 10 **(14)** 11 and 12 and 13 **(15)** Remove duplicates from 14

- 1. Inclusion criteria

Inclusion criteria were: (1) studies reporting an experimental group with a diagnosis of schizophrenia or schizophrenia-spectrum disorder as assessed by recognised standardised diagnostic tool such as Diagnostic and Statistics Manual (DSM) or International Classification of Diseases (ICD); (2) studies reporting a healthy volunteer comparator group; (3) studies reporting PET measures using FDG and report values quantitatively (CMRGlu (mcmol/100g/min or mg/100g/min); Ki values) (4) studies reporting the anatomical location of region from which values were obtained and if applicable normalisation method; (6) studies with full text available in English. Exclusion criteria were (1) studies not reporting direct comparison between designated experimental and control group; (2) studies not providing at the least number of participants per group, mean value per group and standard deviation (or appropriate statistic from which mean and standard deviation can be derived) - reasonable attempts to extract data from graphs will be made; (3) studies reporting FDG uptake using non-standardisable methods included e.g. counts-per-pixel or uptake ratio to occipital lobe.

- 1. Data extraction

The following data were extracted; publication characteristics (authors, year of publication, country of publication, publishing journal); data characteristics (mean and standard deviation (or available statistics) for FDG uptake, unit of measurement of FDG uptake, task or rest measurement, task scores (if applicable), region(s) of interest (ROI), chronicity of illness, medication status, sample size, mean age, sex distribution, duration of illness, diagnostic standard used).

- 1. Equation used to combine mean values for study ROIs within single meta-analysis ROI - 2 study regions

Meta-analysis ROI mean =

*((n(study region 1) x mean(study regions 1))+(n(study region 2) x mean(study region 2)) ÷ (n(study region 1) + n(study region 2))*

- 1. Equation used to combine mean values for study ROIs within single meta-analysis ROI - 3 (or more) study regions

Provisional combined region 1 mean =

*((n(study region 1) x mean(study regions 1))+(n(study region 2) x mean(study region 2)) ÷ (n(study region 1) + n(study region 2))*

Meta-analysis region mean =

*((n(provisional combined region 1) x mean(provisional combined region 1))+(n(study region 3) x mean(study region 3)) ÷ (n(provisional combined region 1) + n(study region 3))*

continues iteratively…

- 1. Equation used to combine standard deviation values for study ROIs within single meta-analysis ROI - 2 study regions

Meta-analysis region SD =

*SQRT ((SD(study region 1^2^) + SD(study region 2^2^) ÷ 2 )*

- 1. Equation used to combine standard deviation values for study ROIs within single meta-analysis ROI - 3 or more study regions

Provisional combined region 1 SD =

*SQRT ((SD(study region 1^2^) + SD(study region 2^2^) ÷ 2 )*

Meta-analysis region SD =

*SQRT ((SD(provisional combined region 1^2^) + SD(study region 3^2^) ÷ 2 )*

continues iteratively…

- 1. Equation used to convert micromole/100mg/min to milligrams/100mg/min

Studies reported CMRGlu using different units. In order to standardise the values for comparison, the following calculation was performed to convert mg/100g/min to micromole/100g/min. Normalised values were not converted.

Value in mg/100g/min x 1000 x (1/molecular weight of fluorodeoxyglucose: 181.1495) = value in micromol/100g/min

Supplementary Table 1:

Table summarising direction of effect for studies excluded from quantitative analysis.

Arrows refer to effect reported by article for stated meta-analysis regions (patients relative to controls):

↑ = significantly higher CMRGlu

↔ = no significant difference in CMRGlu

↓ = significantly lower CMRGlu

1. SUPPLEMENTARY RESULTS - Frontal subgroup analyses
   1. Disease chronicity

The frontal absolute metabolism cohort (Supplementary Fig 4.1) contained 11 studies reporting results for chronic patients, 3 studies reporting results for first-episode patients, and 1 study reporting results for a mixed sample of first episode and chronic patients (mixed). The pooled SMD for chronic studies was -1.07 (95% CI -1.76, -0.38), for first-episode patients it was -0.09 (95% CI [-0.97, 0.79]), and for the single mixed study it was 0.29 (95% CI [-0.23, 0.81]). Test for subgroup differences showed a significant difference between groups (Q=11.58, degrees of freedom (df)=2, p<0.01).

The frontal normalised metabolism cohort (Supplementary Fig 5.1) contained 5 studies of reporting results for chronic patients (chronic studies). The pooled SMD for chronic studies was -0.52 (95% CI [-1.33, 0.29]). 1 study reported results for first-episode patients (FEP studies) with an SMD of -0.05 (95% CI [-0.82, 0.72]). 5 studies reporting a mixed population of chronic and first-episode patients (mixed studies). Pooled SMD for mixed studies was -0.41 (95% CI [-0.92, 0.10]). Test for subgroup differences did not show a significant difference between groups (Q=0.96, df=2, p=0.62).

- 1. Task vs Rest

A mixed-effects subgroup analysis was performed on the frontal absolute and normalised metabolism cohorts with two groups, studies reporting task-based FDG-PET and studies reporting resting state FDG-PET. The frontal absolute metabolism cohort (Supplementary Fig 4.2) contained 3 studies reporting task-based imaging with a pooled SMD of -0.15 (95% CI [-1.49, 1.19]) and 12 studies reporting resting state imaging with a pooled SMD of -0.92 (95% CI [-1.60, -0.23]). Test for subgroup differences did not show a significant difference between groups (Q=3.03, df=1 p=0.08).

The frontal normalised metabolism cohort (Supplementary Fig 5.2) contained 9 studies reporting task-based imaging with a pooled SMD of -0.43 (95% CI [-0.83, -0.03]) and 2 studies reporting resting state imaging with a pooled SMD of -0.48 (95% CI [-1.23, 0.27]). Test for subgroup differences did not show a significant difference between groups (Q=0.01, df=1, p=0.91).

- 1. Medication status

Medication status subgroup analysis of frontal absolute metabolism cohort (Supplementary Fig 4.3) contained five subgroups: drug free (6 studies); medicated (2 studies); mixed (containing medicated and drug free patients) (2 studies); drug naïve (3 studies) and naïve/drug free (2 studies). Test for subgroup differences did not show a significant difference between groups (Q=7.39, df=4, p=0.12). A further analysis was performed pooling the subgroups into two larger subgroups: medicated/mixed (containing 4 studies reporting results for medicated patients and mixed patients cohorts) and naïve/drug free (containing 11 studies reporting results for drug naïve and drug free patients) (Supplementary Fig 4.4); however, no significant difference between these two subgroups was observed (Q=1.89, df=1, p=0.16).

Medication status subgroup analysis of frontal normalised metabolism cohort (Supplementary 5.3) contained five subgroups: drug free (4 studies); medicated (3 studies); mixed (containing medicated and drug free patients) (1 study); drug naïve (1 study) and naïve/drug free (2 studies). Test for subgroup differences showed a significant difference between groups (Q=36.46, df=4, p<0.01). Metabolism for the medicated subgroup was significantly lower than controls (SMD= -1.04; 95% CI [-1.31, -0.78]) while metabolism for other subgroups did not differ from controls. A further analysis was performed pooling the subgroups into two larger subgroups (Supplementary Fig 5.4): medicated/mixed (containing 4 studies reporting results for medicated patients and mixed cohorts) (SMD= -0.84 95% CI [-1.29, -0.38]) and naïve/drug free (containing 7 studies reporting results for drug naïve and drug free patients) (SMD= -0.18 95% CI [-0.43, 0.07]). Mixed effects subgroup analysis found a significant difference between these two subgroups (Q=6.2, df=1, p=0.01).

1. SUPPLEMENTARY RESULTS - meta-analyses for other regions
   1. Temporal lobe metabolism

Pooled results for absolute glucose metabolism in the temporal lobes including data from 11 studies (206 controls, 230 patients) did not demonstrate a statistically significant difference in absolute glucose metabolism (SMD = -0.23; 95% CI = [-0.75; 0.30]; p=0.36) (Supplementary Fig 8) nor did the analysis for normalised glucose metabolism including 8 studies (202 controls, 151 patients) (SMD = 0.35; 95% CI = [-0.03; 0.74]; p=0.07) (Supplementary Fig 9). I^2^ was 73% (moderate heterogeneity) for the analysis of absolute glucose metabolism and 50% (moderate heterogeneity) for the normalised analysis. No outliers were detected for either the absolute or normalised temporal metabolism cohorts.

- 1. Parietal lobe metabolism

Data for absolute glucose metabolism in the parietal lobes from 12 studies (217 controls, 255 patients) were included in the analysis. Pooled results did not demonstrate a statistically significant difference in absolute (SMD = -0.45; 95% CI = [-1.10; 0.20]; p=0.16) (Supplementary Fig 10). I^2^ was 78% (substantial heterogeneity). Studies by Huret *et al.* (1991) and Siegel *et al.* (1993) were identified as outliers. Further random-effects analysis was conducted with these studies removed finding a pooled SMD -0.3255 (95% CI [-0.76; 0.11]). This difference was not statistically significant (p=0.13).

Pooled results for normalised glucose metabolism in the parietal lobe including results from 6 studies (173 controls, 109 patients) did not demonstrate a statistically significant difference in normalised glucose metabolism (SMD = 0.17; 95% CI = [-0.45; 0.80]; p=0.51) (Supplementary Fig 11). I^2^ was 70% (substantial heterogeneity) but no outliers were detected.

Occipital lobe metabolism

Pooled results for absolute glucose metabolism in the occipital lobe, from 12 studies (203 controls, 207 patients) did not demonstrate a statistically significant difference in absolute metabolism relative to controls (SMD = -0.55; 95% CI = [-1.12; 0.01]; p=0.06) (Supplementary Fig 12) nor did the analysis for normalised glucose metabolism, including data from 5 studies (110 controls, 87 patients) (SMD = 0.20; 95% CI = [-0.48; 0.89]; p=0.46) (Supplementary Fig 13). For analysis of absolute glucose metabolism, I^2^ was 77% (substantial heterogeneity). For analysis of normalised glucose metabolism, I^2^ was 59% (moderate heterogeneity). No outliers were detected for either cohort.

- 1. Thalamic metabolism

Pooled results for absolute glucose metabolism in the thalamus from 9 studies (176 controls, 229 patients) did not demonstrate a statistically significant difference in absolute metabolism relative to controls (SMD = -0.08; 95% CI = [-0.48; 0.32]; p=0.66) (Supplementary Fig 14). I^2^ was 69% (moderate heterogeneity). A study by Siegel *et al.* (1993) was identified as an outlier. Further random-effects analysis was conducted with this study removed finding a pooled SMD -0.22 (95% CI [-0.51; 0.06]; I^2^=13%). This difference was not statistically significant (p=0.11).

The analysis for normalised glucose metabolism including data from 6 studies (127 controls, 115 patients) (SMD = -0.34; 95% CI = [-0.90; 0.22]; p=0.18) (Figure 15) did not find a significant difference between patients and controls. I^2^ was 61% (moderate heterogeneity) for the normalised cohort and no outliers were detected.

SUPPLEMENTARY TABLES

Supplementary Table 1:

Table showing details of full texts not included in meta-analysis with reason for exclusion and direction of reported effect (patient relative to controls)

| Authors | Title | Publication year | Exclusion reason | Reported effect  FRONTAL | Reported effect  PARIETAL | Reported effect  TEMPORAL | Reported effect  OCCIPITAL | Reported effect  BASAL GANGLIA | Reported effect  THALAMUS |
| --- | --- | --- | --- | --- | --- | --- | --- | --- | --- |
| Buchsbaum et al. | Cerebral glucography with positron tomography. Use in normal subjects and in patients with schizophrenia | 1982 | rCMRglc not reported - counts per pixel only | ↓ | not reported | not reported | not reported | not reported | not reported |
| Buchsbaum et al. | Positron emission tomographic image measurement in schizophrenia and affective disorders | 1984 | duplicate of Buchsbaum 1984 "Anteropost…" | n/a | n/a | n/a | n/a | n/a | n/a |
| Jernigan et al. | 18Fluorodeoxyglucose PET in schizophrenia | 1985 | rCMRglc not reported - counts per pixel only | ↔ | not reported | ↑ | ↔ | not reported | not reported |
| Cohen et al. | Dysfunction in a prefrontal substrate of sustained attention in schizophrenia | 1987 | rCMRglc not reported - rGMR difference reported | ↓ | not reported | not reported | not reported | not reported | not reported |
| Buchsbaum et al. | Positron emission tomography studies of basal ganglia and somatosensory cortex neuroleptic drug effects: Differences between normal controls and schizophrenic patients | 1987 | excluded from frontal as overlaps with Buschbaum 1984 | n/a | n/a | n/a | n/a | n/a | n/a |
| Szechtman et al. | Effect of neuroleptics on altered cerebral glucose metabolism in schizophrenia | 1988 | rCMRglc not reported -counts per pixel only | ↑ | not reported | not reported | not reported | not reported | not reported |
| Guich et al. | Effect of attention on frontal distribution of delta activity and cerebral metabolic rate in schizophrenia | 1989 | statistics not extractable - mean and SD not reported | ↓ | not reported | not reported | not reported | not reported | not reported |
| Cleghorn et al. | Apomorphine effects on brain metabolism in neuroleptic-naive schizophrenic patients | 1991 | rCMRglc not reported - whole brain slices | n/a | n/a | n/a | n/a | n/a | n/a |
| Biver et al. | No hypofrontality in schizophrenia demonstrated by positron emission tomography | 1992 | In French, full-text not available | ↔ (according to abstract) | not reported | not reported | not reported | not reported | not reported |
| Siegel et al. | Cortical-striatal-thalamic circuits and brain glucose metabolic activity in 70 unmedicated male schizophrenic patients | 1993 | excluded from frontal and occipital analysis as overlaps with Buchsbaum 1990, 1992 | n/a | n/a | n/a | n/a | n/a | n/a |
| Clark et al. | Metabolic subtypes in patients with schizophrenia | 1993 | statistics not extractable - SD not given | ↓ | ↓ | ↔ | not reported | ↔ | ↔ |
| Hazlett et al. | Reduced regional brain glucose metabolism assessed by positron emission tomography in electrodermal nonresponder schizophrenics: A pilot study | 1993 | statistics not extractable - control group SD not given | ↔ | not reported | not reported | not reported | not reported | ↔ |
| Guenther et al. | Diminished cerebral metabolic response to motor stimulation in schizophrenics: A PET study | 1993 | rCMRglc not reported - % activation from baseline | ↓ | not reported | not reported | not reported | ↑ | ↑ |
| Siegel et al. | Glucose metabolic correlates of continuous performance test performance in adults with a history of infantile autism, schizophrenics, and controls | 1995 | rCMRglc not reported - non standard ratio calculation | ↓ | not reported | not reported | not reported | not reported | not reported |
| Schroder et al. | Cerebral metabolic activity correlates of subsyndromes in chronic schizophrenia | 1996 | rCMRglc not reported - factor analysis | ↓ | not reported | not reported | not reported | not reported | not reported |
| Haznedar et al. | Decreased anterior cingulate gyrus metabolic rate in schizophrenia | 1997 | rCMRglc not reported - relative change only | ↓ | not reported | not reported | not reported | not reported | not reported |
| Wong et al. | Positron emission tomography in male violent offenders with schizophrenia. | 1997 | rCMRglc not reported - fdg uptake only | not reported | not reported | ↓ | not reported | not reported | not reported |
| Cohen et al. | Abnormalities in the distributed network of sustained attention predict neuroleptic treatment response in schizophrenia | 1998 | excluded from frontal analysis as overlaps with Cohen 1997 | n/a | n/a | n/a | n/a | n/a | n/a |
| Buchsbaum et al. | MRI white matter diffusion anisotropy and PET metabolic rate in schizophrenia | 1998 | rCMRglc not reported - correlation coeffcient only | ↓ | not reported | not reported | not reported | not reported | not reported |
| Buchsbaum et al. | Visualizing fronto-striatal circuitry and neuroleptic effects in schizophrenia | 1999 | rCMRglc not reported - correlation coefficient only | ↓ | not reported | not reported | not reported | not reported | not reported |
| Lauer et al. | Disturbed neural circuits in a subtype of chronic catatonic schizophrenia demonstrated by F-18-FDG-PET and F-18-DOPA-PET | 2001 | small sample size - patient group n=3 | ↔ | not reported | ↓ | not reported | not reported | ↔ |
| Buchsbaum et al. | Differential metabolic rates in prefrontal and temporal Brodmann areas in schizophrenia and schizotypal personality disorder | 2002 | rCMRglc not reported - relative GMR only | ↓ | not reported | ↓ | not reported | not reported | not reported |
| Buchsbaum et al. | Kraepelinian and non-Kraepelinian schizophrenia subgroup differences in cerebral metabolic rate | 2002 | rCMRglc not reported - relative GMR only | ↓ | ↓ | ↓ | ↓ | not reported | not reported |
| Potkin et al. | A PET study of the pathophysiology of negative symptoms in schizophrenia | 2002 | rCMRglc not reported -MNI coordinates only | not reported | not reported | not reported | not reported | not reported | not reported |
| Desco et al. | Cerebral metabolic patterns in chronic and recent-onset schizophrenia | 2003 | rCMRglc not reported -MNI coordinates only | not reported | not reported | ↑ | ↓ | not reported | not reported |
| Kopecek et al. | Regional glucose uptake (18-FDG-PET) in patients with the 1st episode of schizophrenia | 2003 | In Polish, text not found |  |  |  |  |  |  |
| Hazlett et al. | Abnormal glucose metabolism in the mediodorsal nucleus of the thalamus in schizophrenia | 2004 | rCMRglc not reported - relative GMR only | not reported | not reported | not reported | not reported | not reported | ↓ |
| Horacek et al. | Resting regional brain metabolism in patients with schizophrenia. 18-FDG PET study | 2004 | rCMRglc not reported -MNI coordinates only | ↑ | not reported | not reported | not reported | not reported | not reported |
| Haznedar et al. | Cingulate gyrus volume and metabolism in the schizophrenia spectrum | 2004 | rCMRglc not reported - relative GMR only | ↓ | not reported | not reported | not reported | not reported | not reported |
| Soyka et al. | Hypermetabolic pattern in frontal cortex and other brain regions in unmedicated schizophrenia patients: Results from a FDG-PET study | 2005 | rCMRglc not reported - ratio to occipital lobe only | ↑ | not reported | not reported | not reported | not reported | not reported |
| Park et al. | Cortical surface-based analysis of 18F-FDG PET: Measured metabolic abnormalities in schizophrenia are affected by cortical structural abnormalities | 2006 | rCMRglc not reported -MNI coordinates only | ↓ | not reported | ↑ | not reported | not reported | not reported |
| Buchsbaum et al. | Relative glucose metabolic rate higher in white matter in patients with schizophrenia | 2007 | rCMRglc not reported - relative GMR only | ↑ | not reported | ↑ | not reported | not reported | not reported |
| Molina et al. | Marked hypofrontality in clozapine-responsive patients | 2007 | rCMRglc not reported -MNI coordinates only | ↓ | not reported | not reported | not reported | not reported | not reported |
| Buchsbaum et al. | FDG-PET and MRI imaging of the effects of sertindole and haloperidol in the prefrontal lobe in schizophrenia | 2009 | No comparison possible - no control group | n/a | n/a | n/a | n/a | n/a | n/a |
| Park et al. | Medial prefrontal default-mode hypoactivity affecting trait physical anhedonia in schizophrenia | 2009 | rCMRglc not reported - MNI coordinates only | ↓ | not reported | not reported | not reported | not reported | not reported |
| Park et al. | Anhedonia and ambivalence in schizophrenic patients with fronto-cerebellar metabolic abnormalities: A fluoro-D-glucose positron emission tomography study | 2009 | rCMRglc not reported - no quantification | ↓ | not reported | not reported | not reported | not reported | not reported |
| Fernandez-Egea et al. | 18FDG PET study of amygdalar activity during facial emotion recognition in schizophrenia | 2010 | rCMRglc not reported -MNI coordinates only | n/a | n/a | n/a | n/a | n/a | n/a |
| Parkar et al. | Are the effects of cannabis dependence on glucose metabolism similar to schizophrenia? An FDG PET understanding | 2011 | rCMRglc not reported - ratio to occipital lobe | ↔ | ↔ | ↔ | ↔ | ↔ | ↔ |
| Horga et al. | Brain metabolism during hallucination-like auditory stimulation in schizophrenia | 2014 | rCMRglc not reported -MNI coordinates only | n/a | n/a | n/a | n/a | n/a | n/a |
| Shinto et al. | Hyperfrontality as seen on FDG PET in unmedicated schizophrenia patients with positive symptoms | 2014 | rCMRglc not reported - ratio to occipital lobe | ↑ | not reported | not reported | not reported | not reported | not reported |
| Choudhary et al. | F-18 fluorodeoxyglucose positron emission tomography study of impaired emotion processing in first episode schizophrenia | 2015 | rCMRglc not reported -MNI coordinates only | ↓ | not reported | not reported | not reported | ↑ | not reported |
| Bralet et al. | FDG-PET scans in patients with Kraepelinian and non-Kraepelinian schizophrenia | 2016 | rCMRglc not reported - relative GMR only | ↓ | not reported | ↓ | not reported | not reported | ↓ |
| Kim et al. | Altered interregional correlations between serotonin transporter availability and cerebral glucose metabolism in schizophrenia: A high-resolution PET study using [11C]DASB and [18F]FDG. | 2017 | not reported | ↓ | ↓ | ↓ | ↓ | not reported | not reported |
| Mitelman et al. | Increased white matter metabolic rates in autism spectrum disorder and schizophrenia | 2018 | rCMRglc not reported - relative GMR only | ↓ (grey matter) ↑ (white matter) | not reported | not reported | not reported | not reported | not reported |
| Mitelman et al. | Positron emission tomography assessment of cerebral glucose metabolic rates in autism spectrum disorder and schizophrenia | 2018 | rCMRglc not reported - relative change only | ↓ | not reported | ↓ | not reported | ↓ | ↓ |

Supplementary Table 2:

Table showing number of full text articles excluded from quantitative analysis for each meta-analysis region and % of excluded studies reporting given direction of effect for regional glucose metabolism


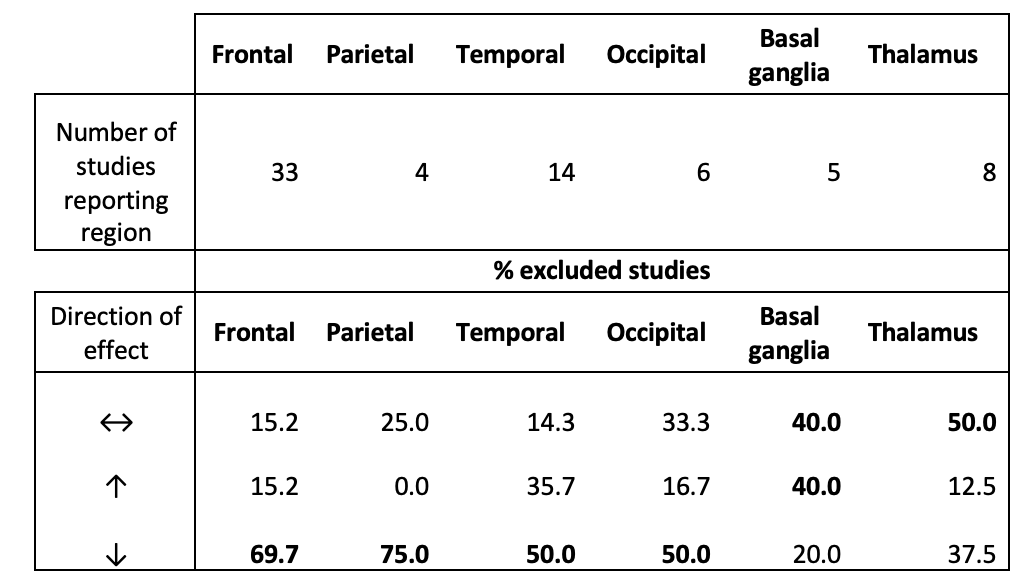


SUPPLEMENTARY FIGURES

**Supplementary Fig 1 Frontal absolute metabolism influence analysis:**

**Leave one out analysis demonstrating effect of omitting studies on effect size and heterogeneity**


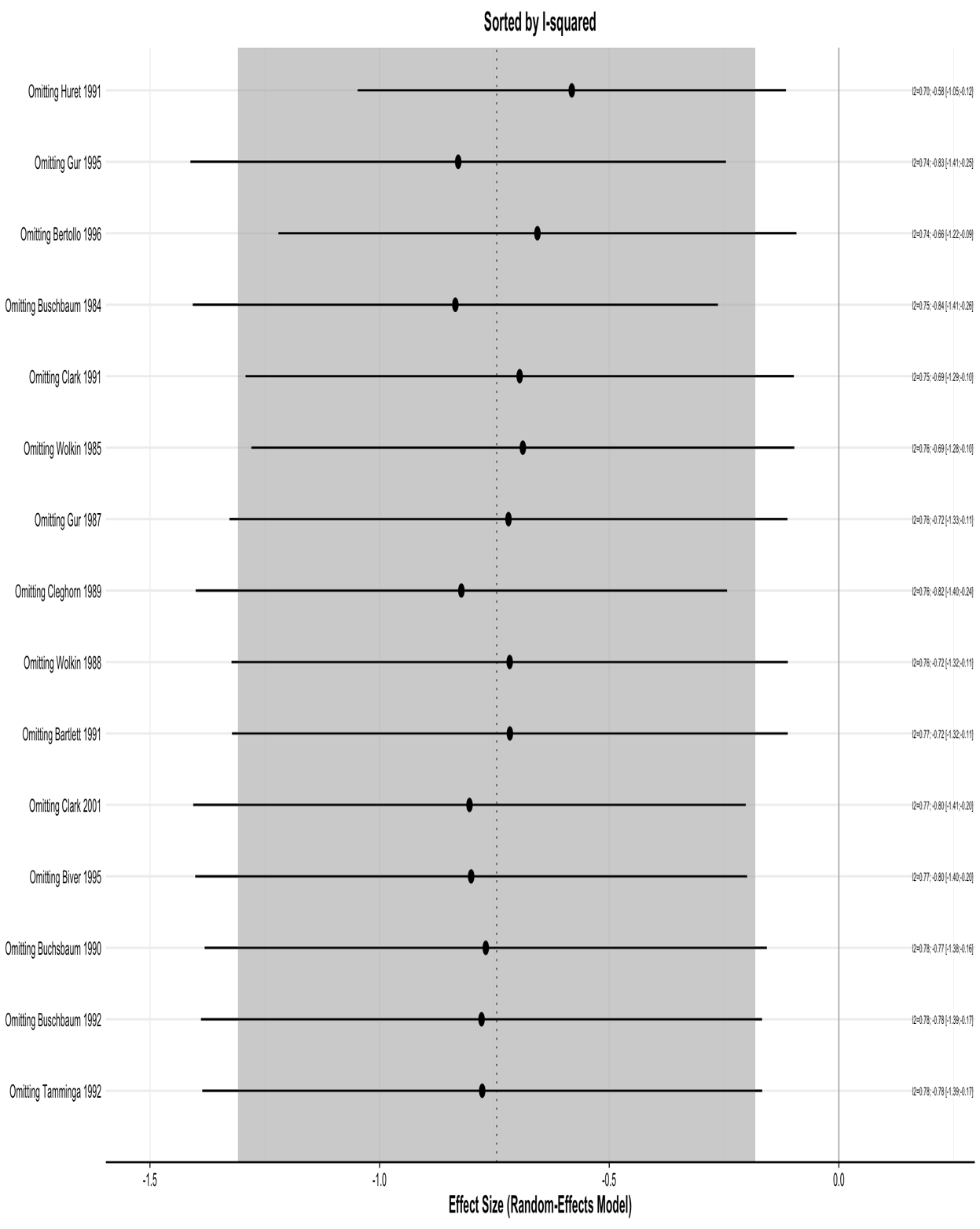

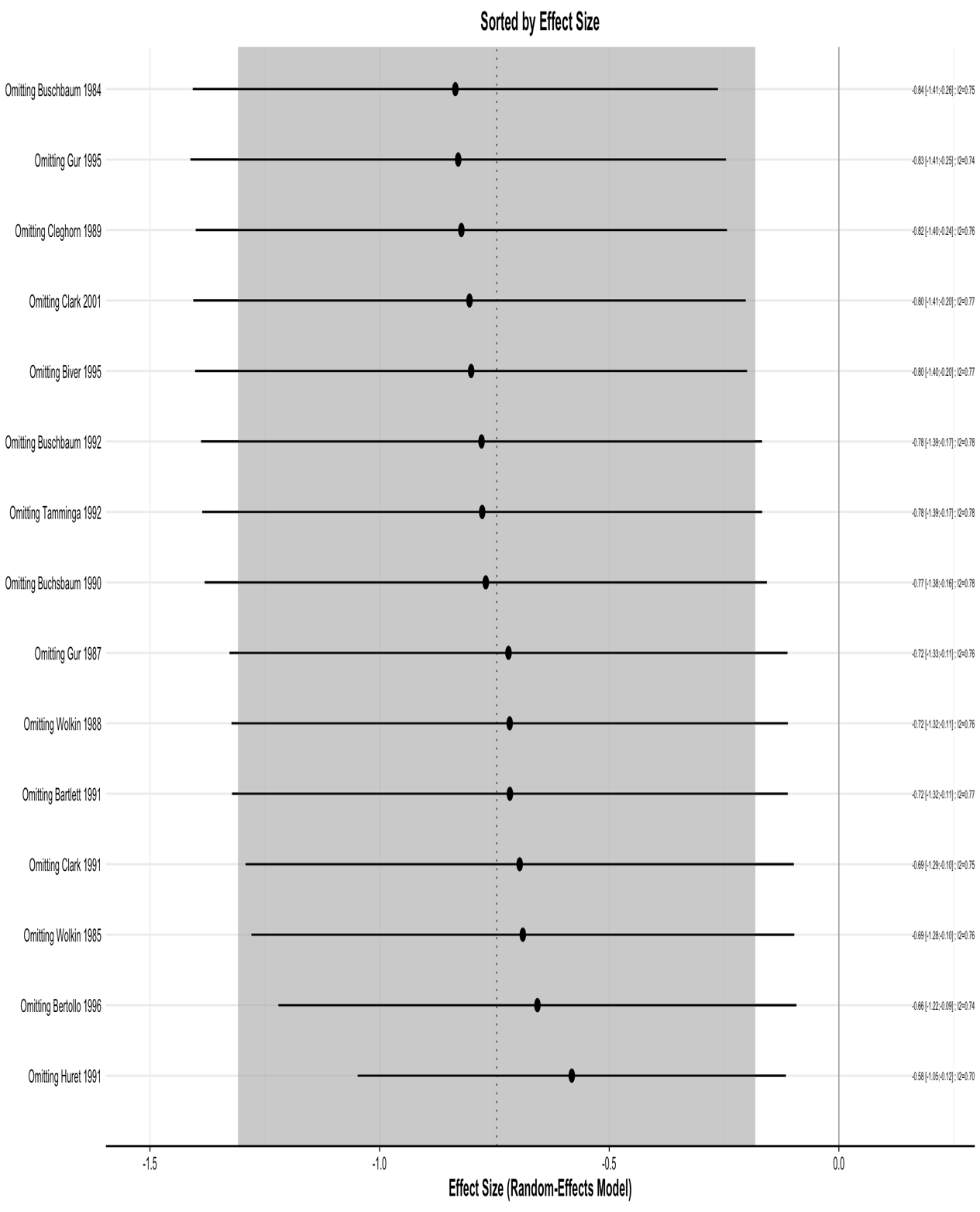

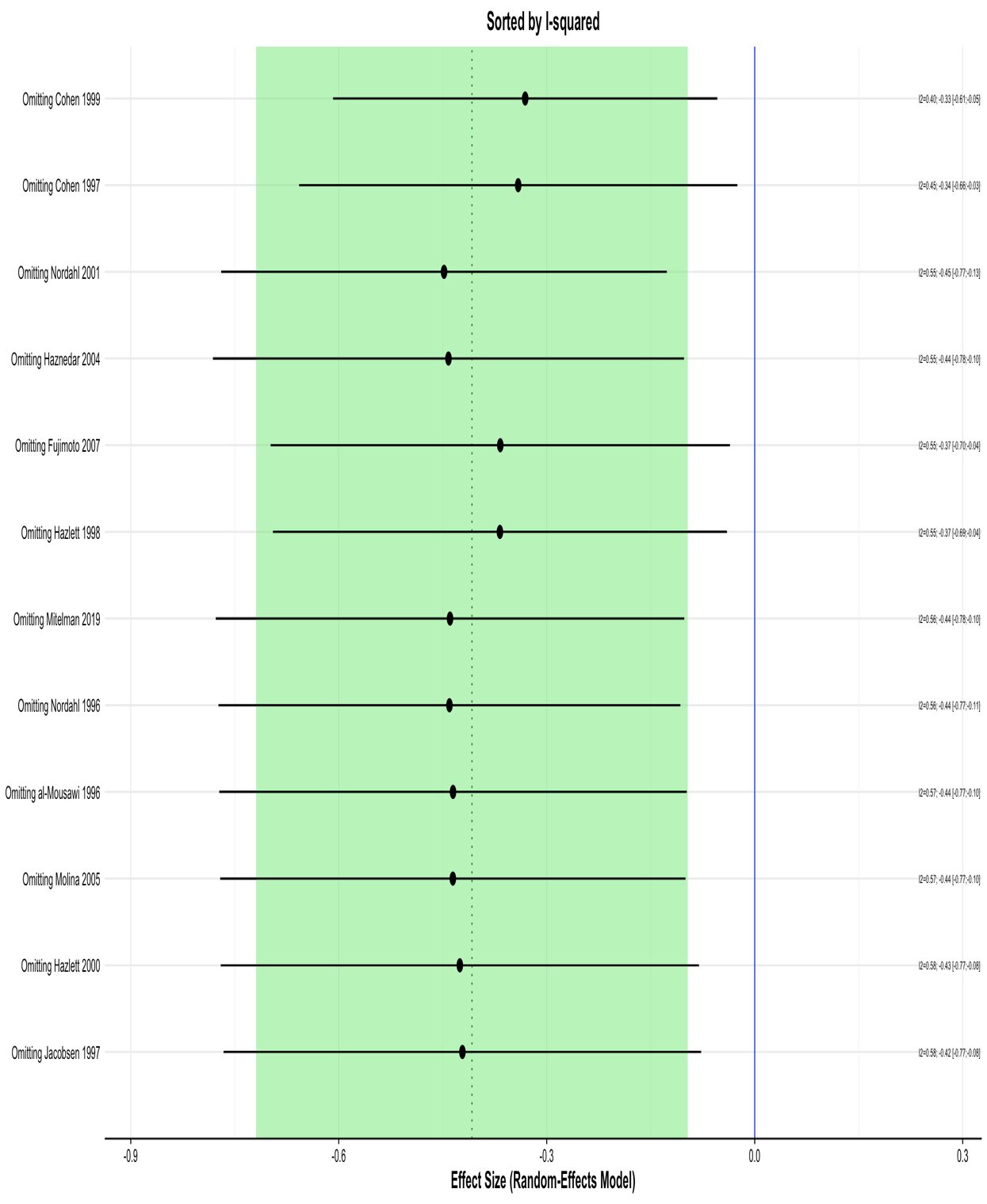

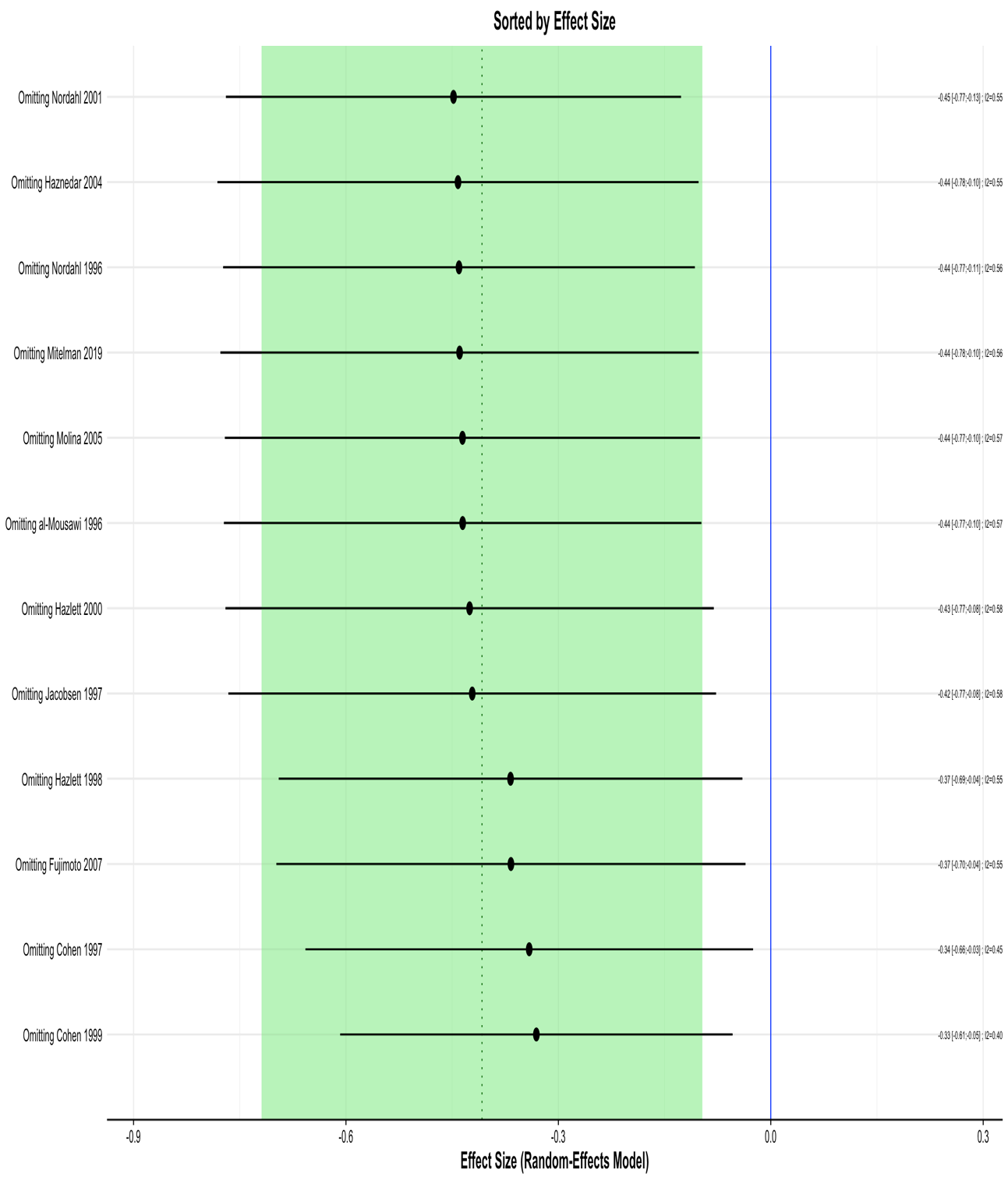


**Supplementary Fig 2 Frontal normalised metabolism influence analysis:**

**Leave one out analysis demonstrating effect of omitting studies on effect size and heterogeneity**

**Supplementary Fig 4 - Frontal absolute subgroup analysis**

**Fig 4.1 – Mixed effects model assessing effect of disease chronicity on pooled SMD**


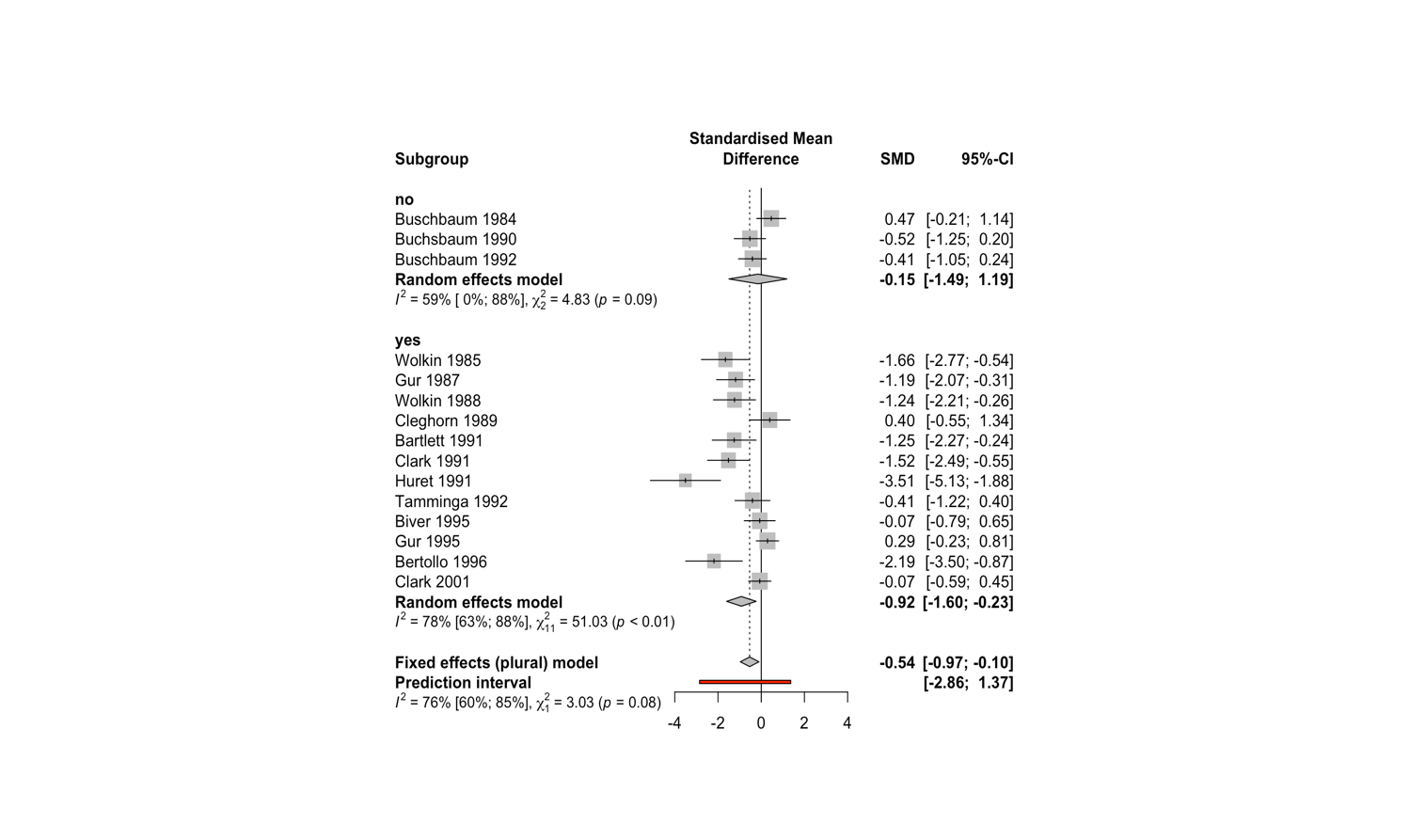


**Fig 4.2 – Mixed effects model assessing effect of task vs rest on pooled SMD**

**Fig 4.3 – Mixed effects model assessing effect of medication status on pooled SMD**


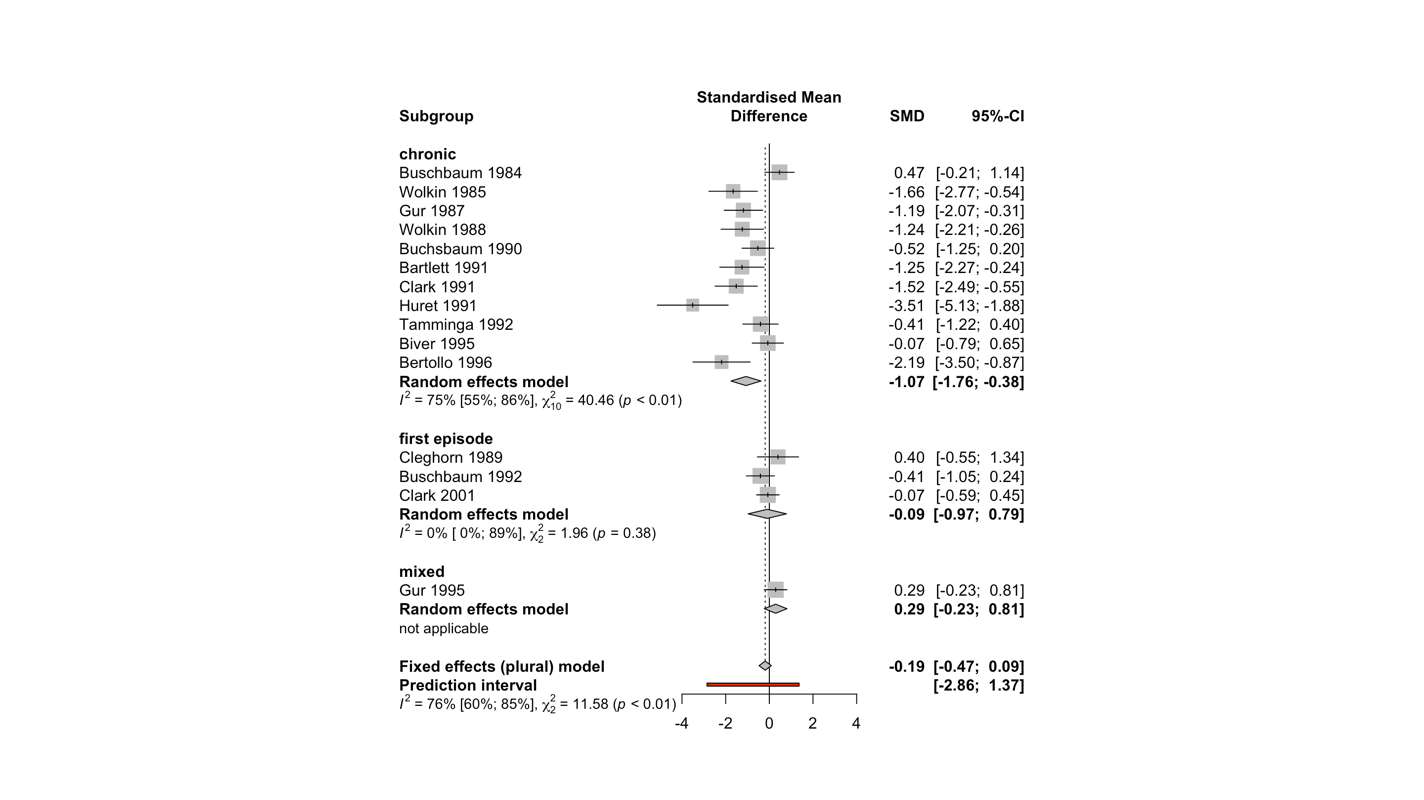

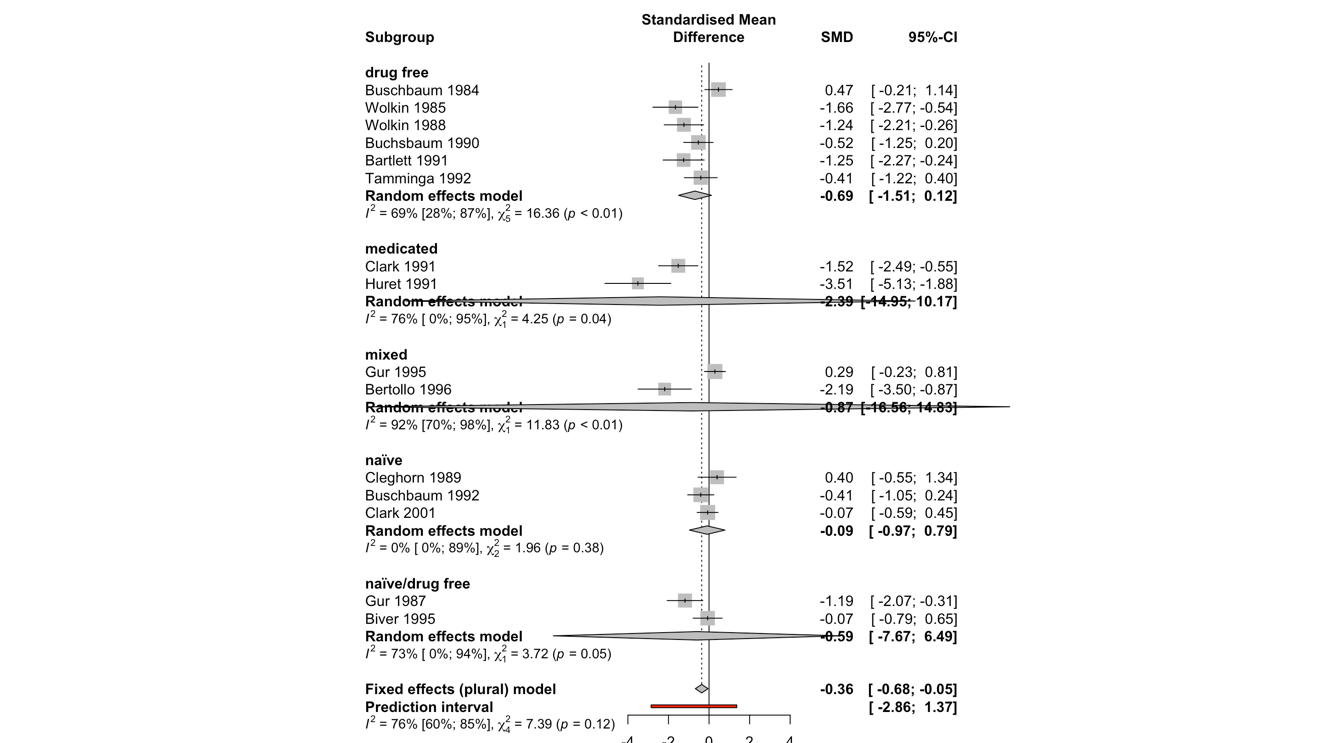

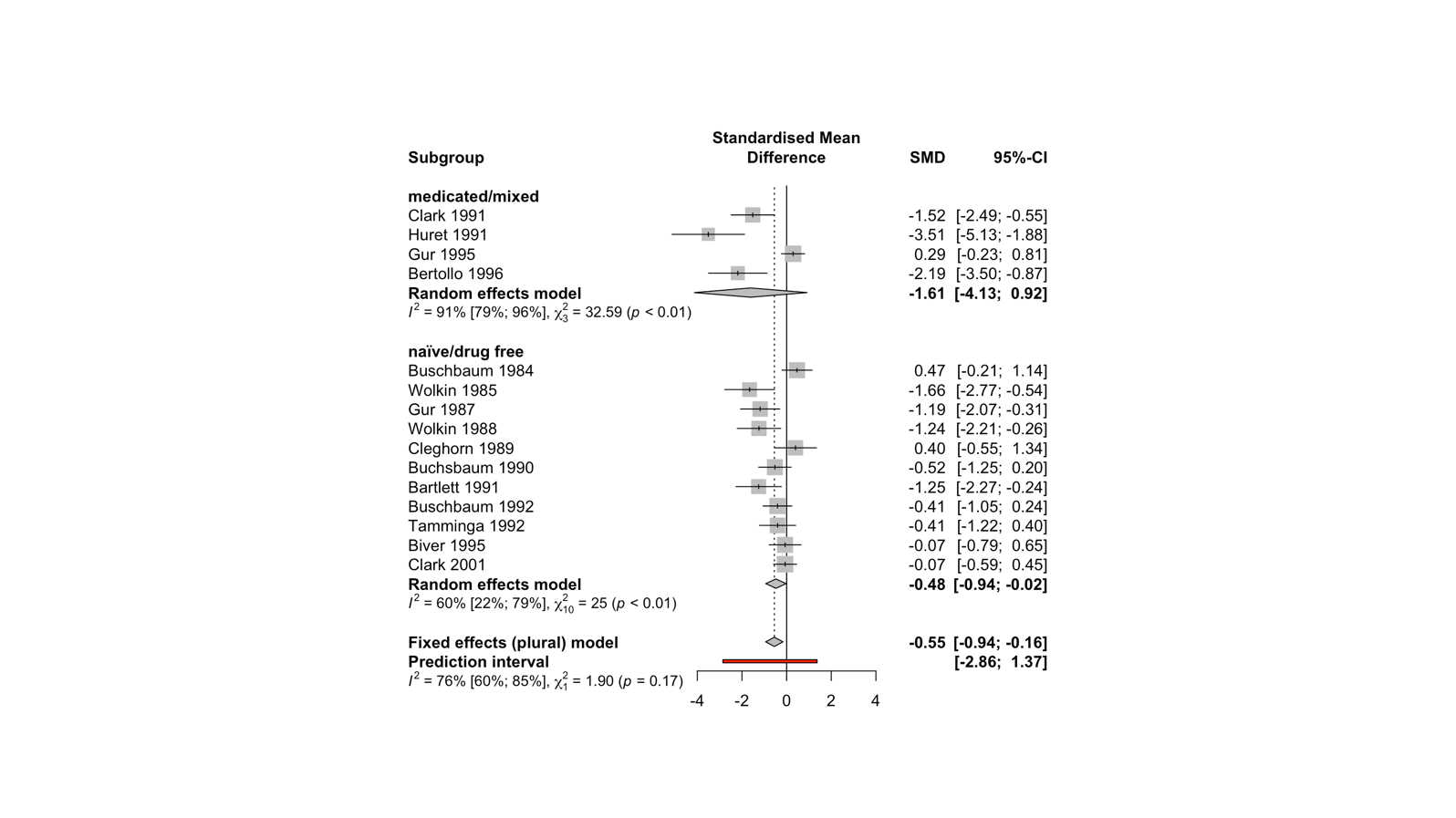


**Fig 4.4 – Mixed effects model assessing effect of medication status (with simplified subgroups) on pooled SMD**

**Supplementary Fig 3 Frontal absolute metabolism sensitivity analysis:**

**Forest plot showing results of random effects meta-analysis omitting studies contributing most significantly to heterogeneity (Huret 1991; Gur 1995)**


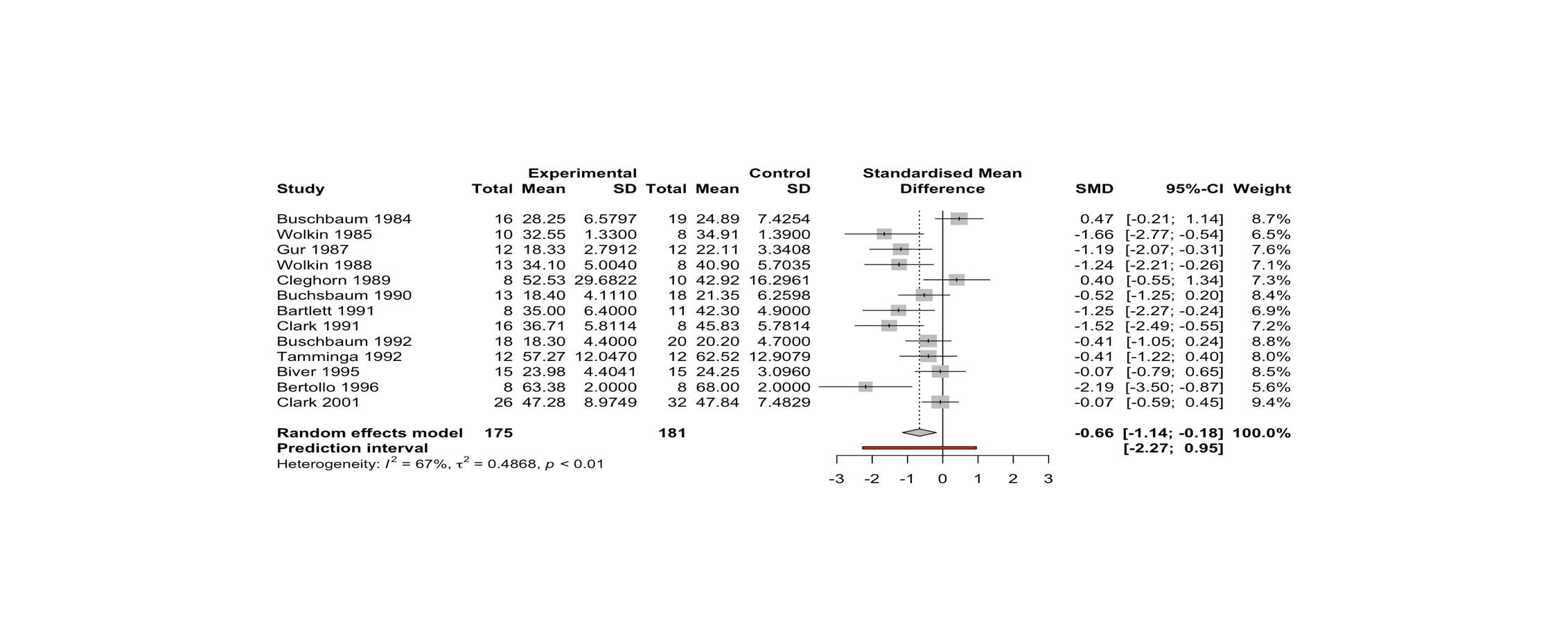


**Supplementary Fig 5 - Frontal normalised subgroup analysis**

**Fig 5.1 – Mixed effects model assessing effect of disease chronicity on pooled SMD**

**Fig 5.2 – Mixed effects model assessing effect of task vs rest on pooled SMD**


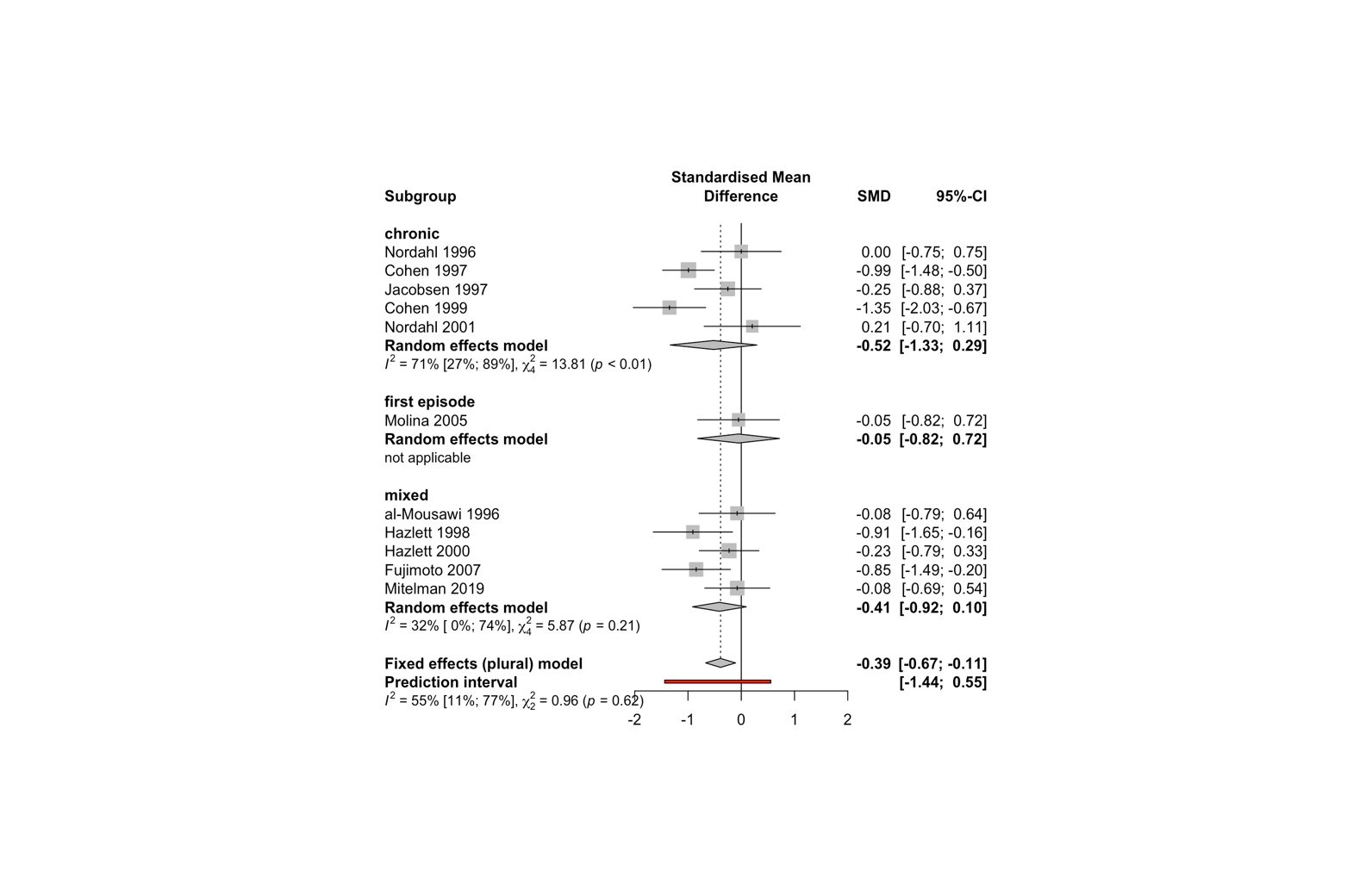

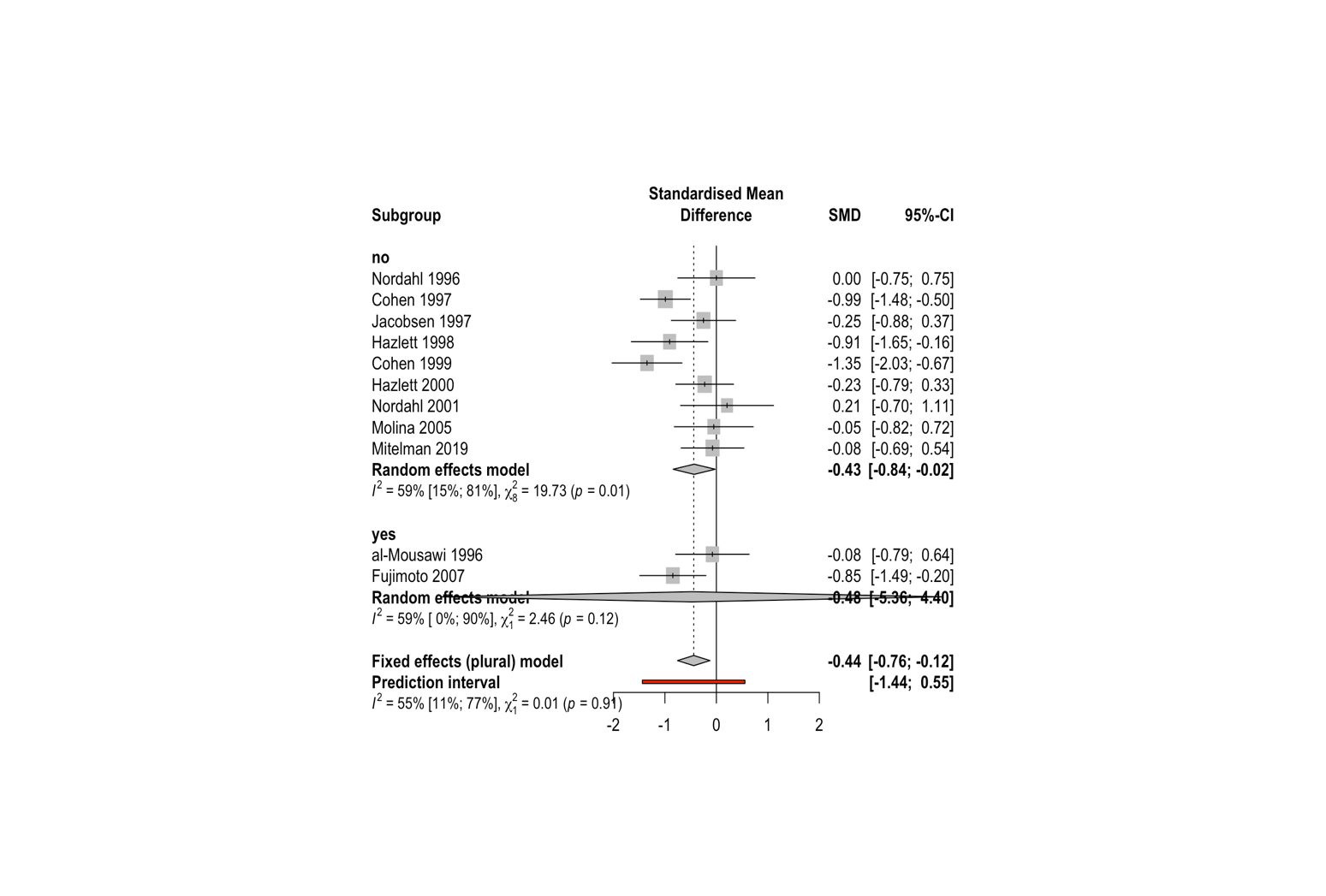


**Fig 5.3 – Mixed effects model assessing effect of medication status on pooled SMD**


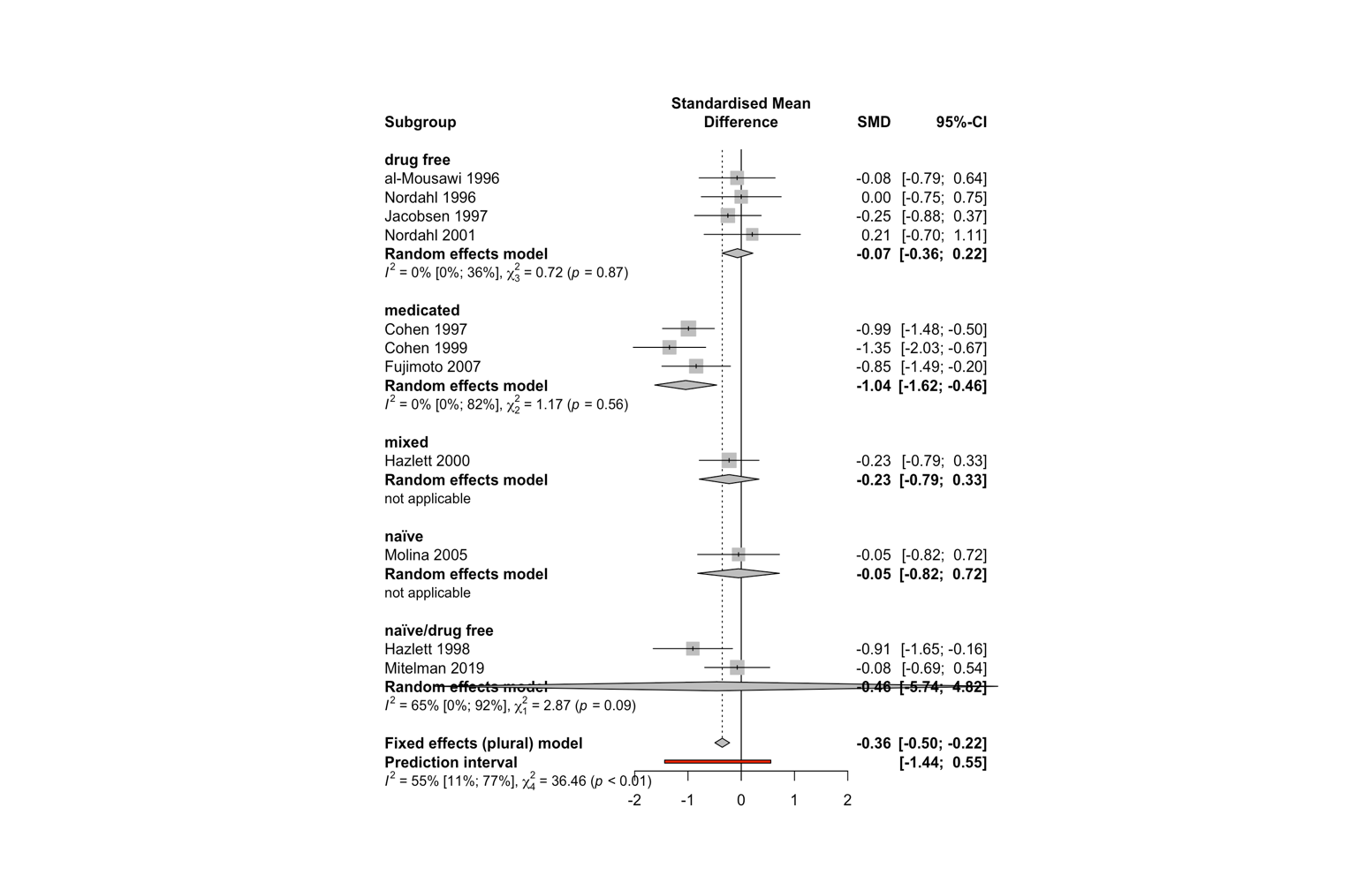


**Fig 5.4– Mixed effects model assessing effect of medication status (with simplified subgroups) on pooled SMD**


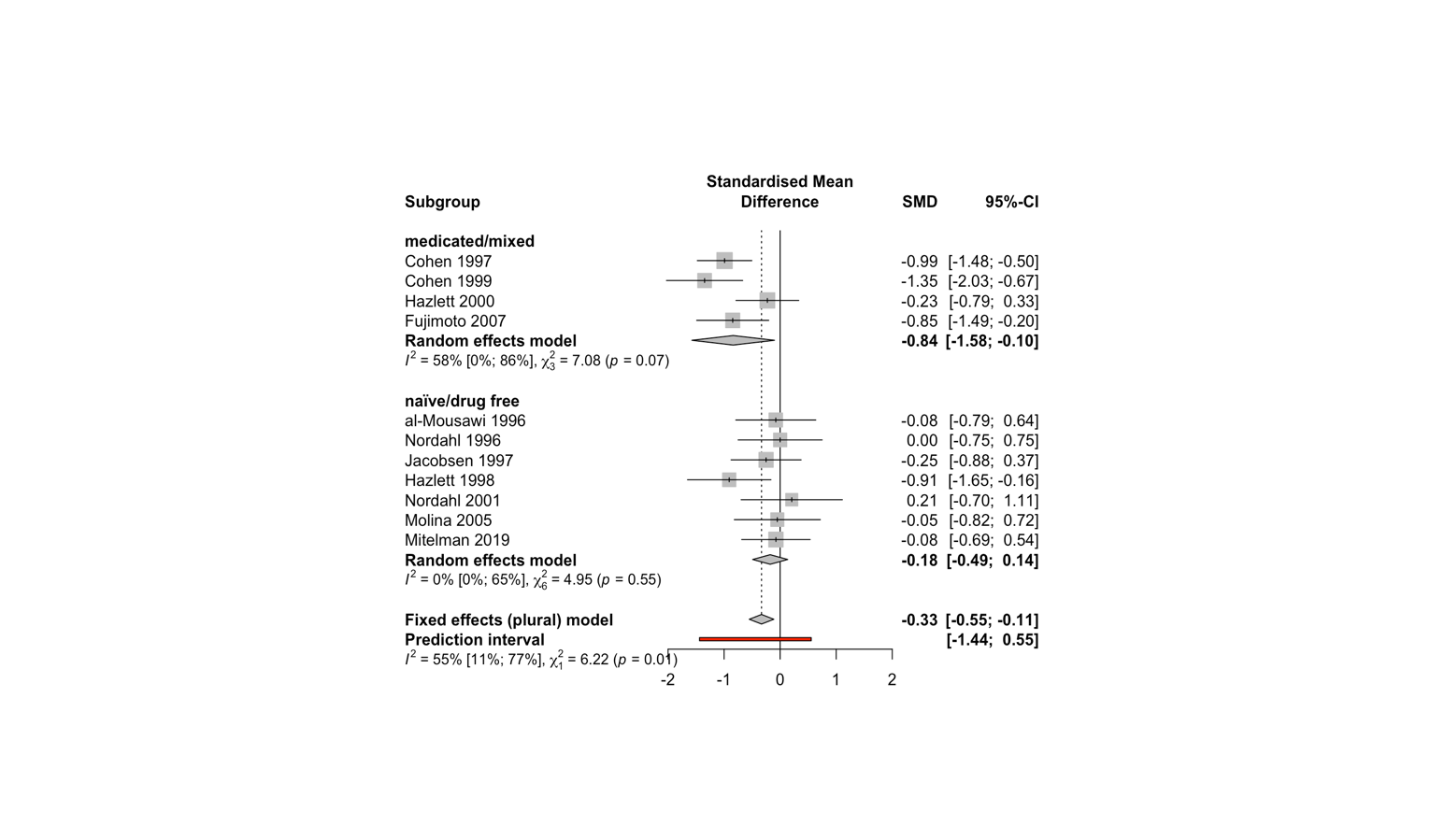

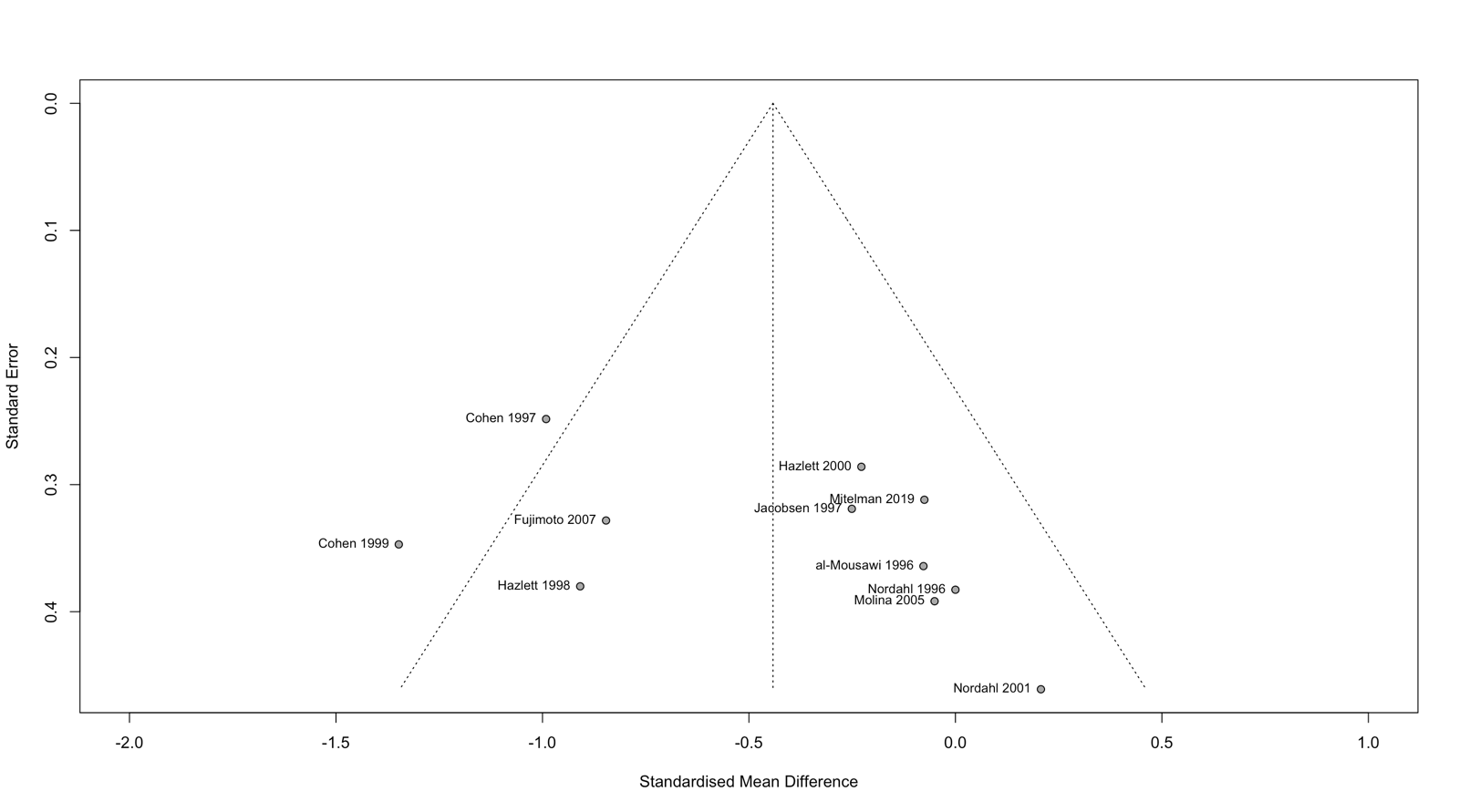


**Fig 5.6 – Funnel plot for frontal normalised cohort - to assess presence of publication bias through small study effects**


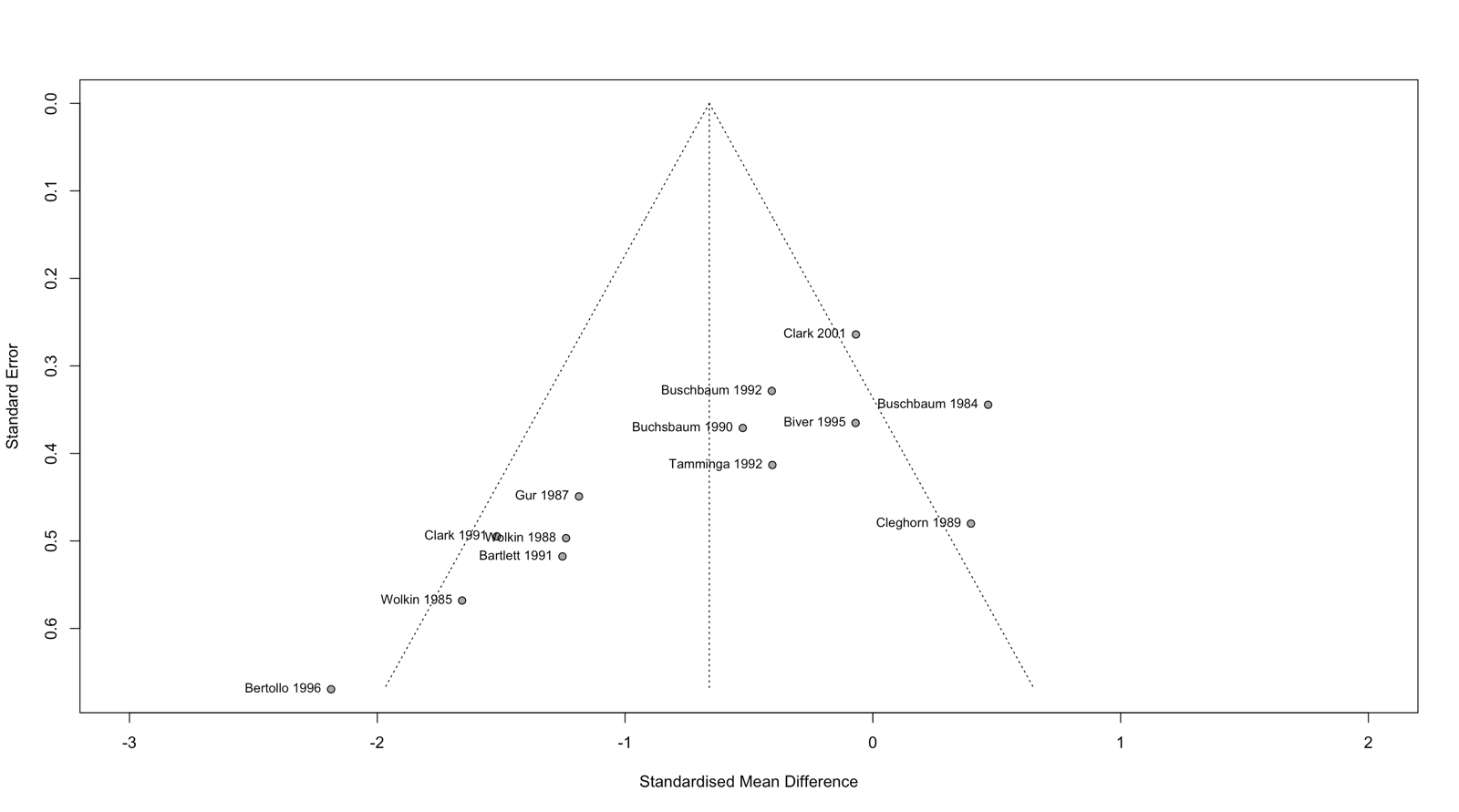


**Fig 5.5 – Funnel plot for frontal absolute cohort (with outliers excluded) - to assess presence of publication bias through small study effects**

**Supplementary Fig 6.1 Meta-analysis of studies reporting basal ganglia absolute CMRGlu:**

**Forest plot showing results of random-effects meta-analysis**

**Supplementary Fig 7 Meta-analysis of studies reporting basal ganglia normalised CMRGlu:**

**Forest plot showing results of random-effects meta-analysis**


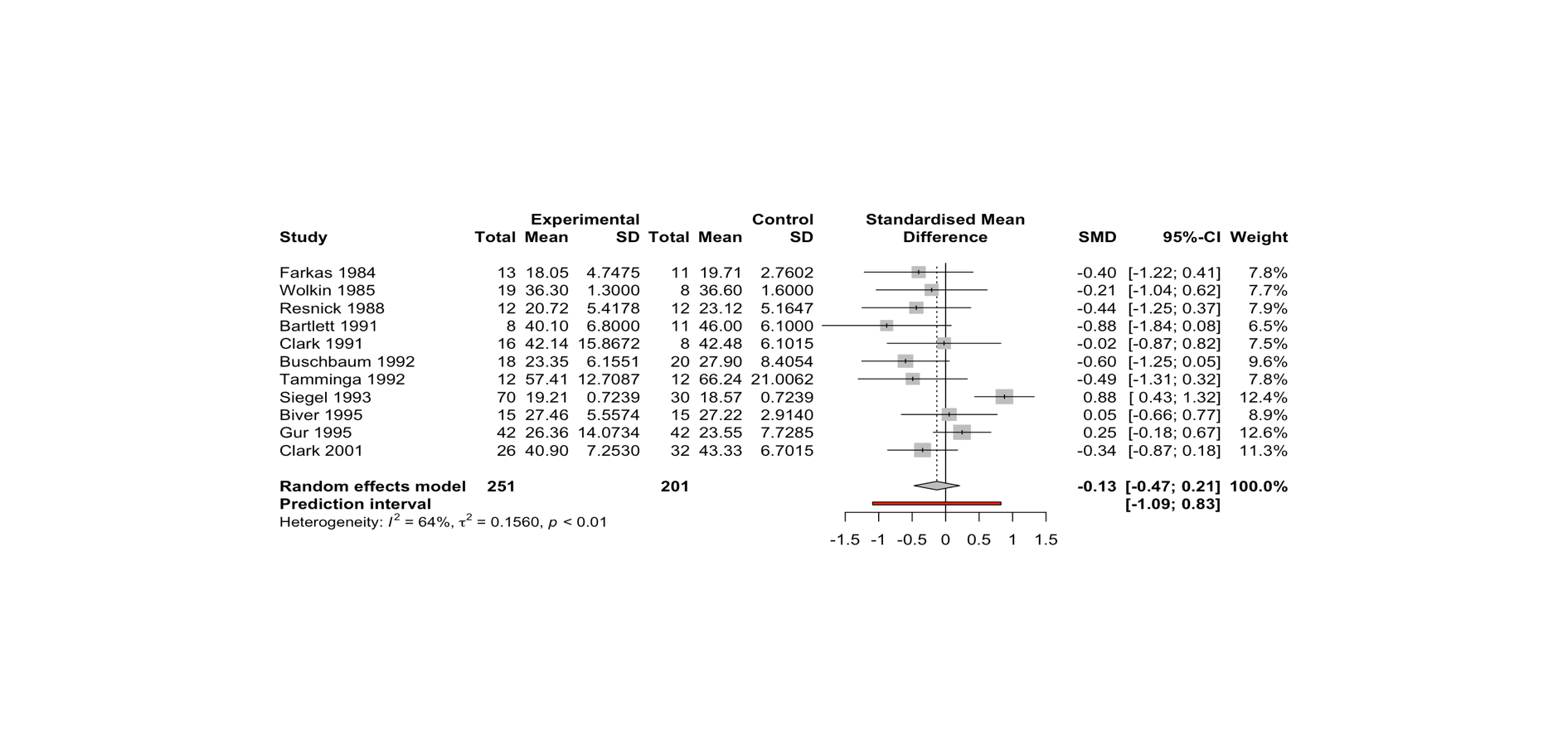

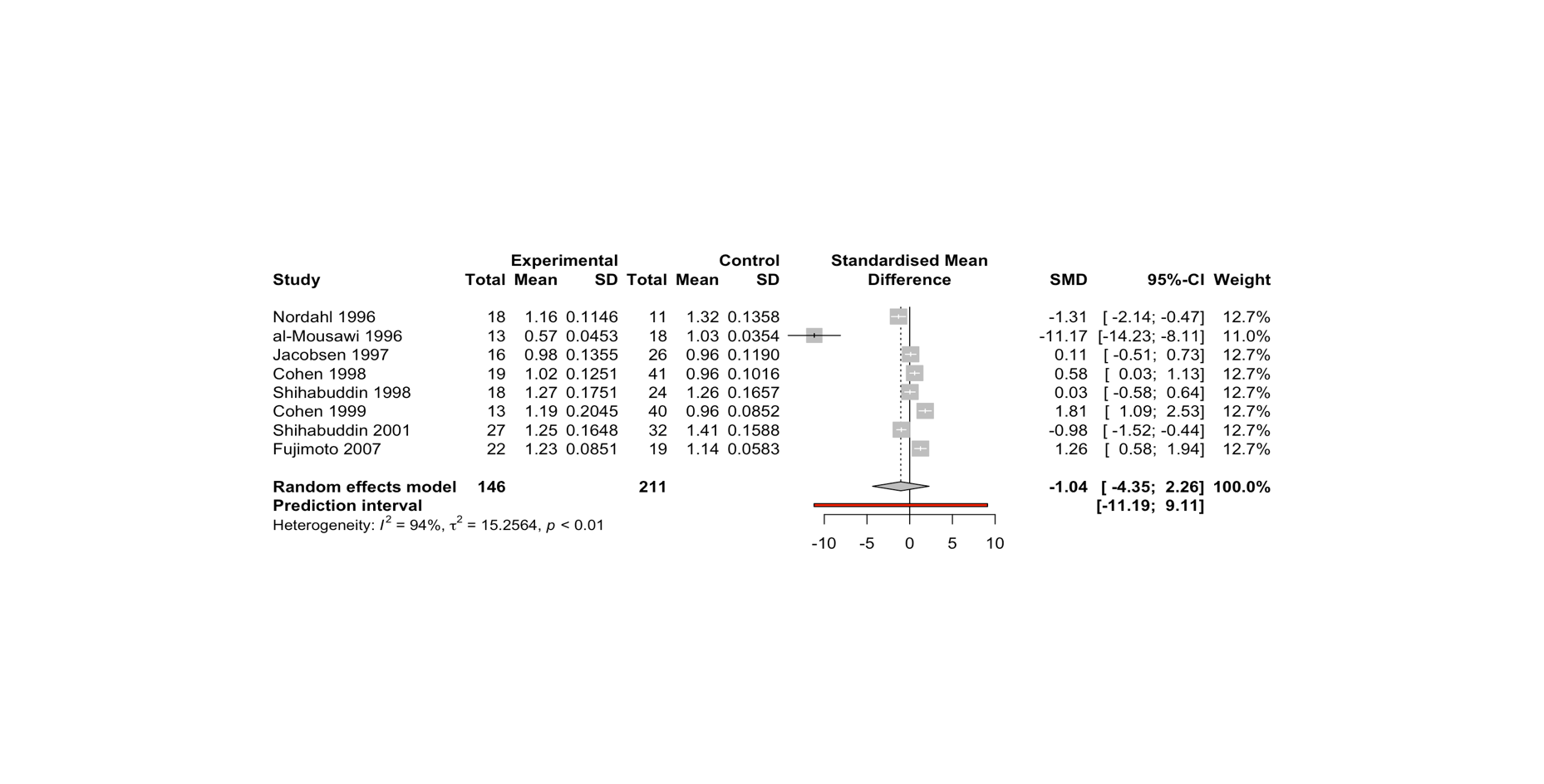

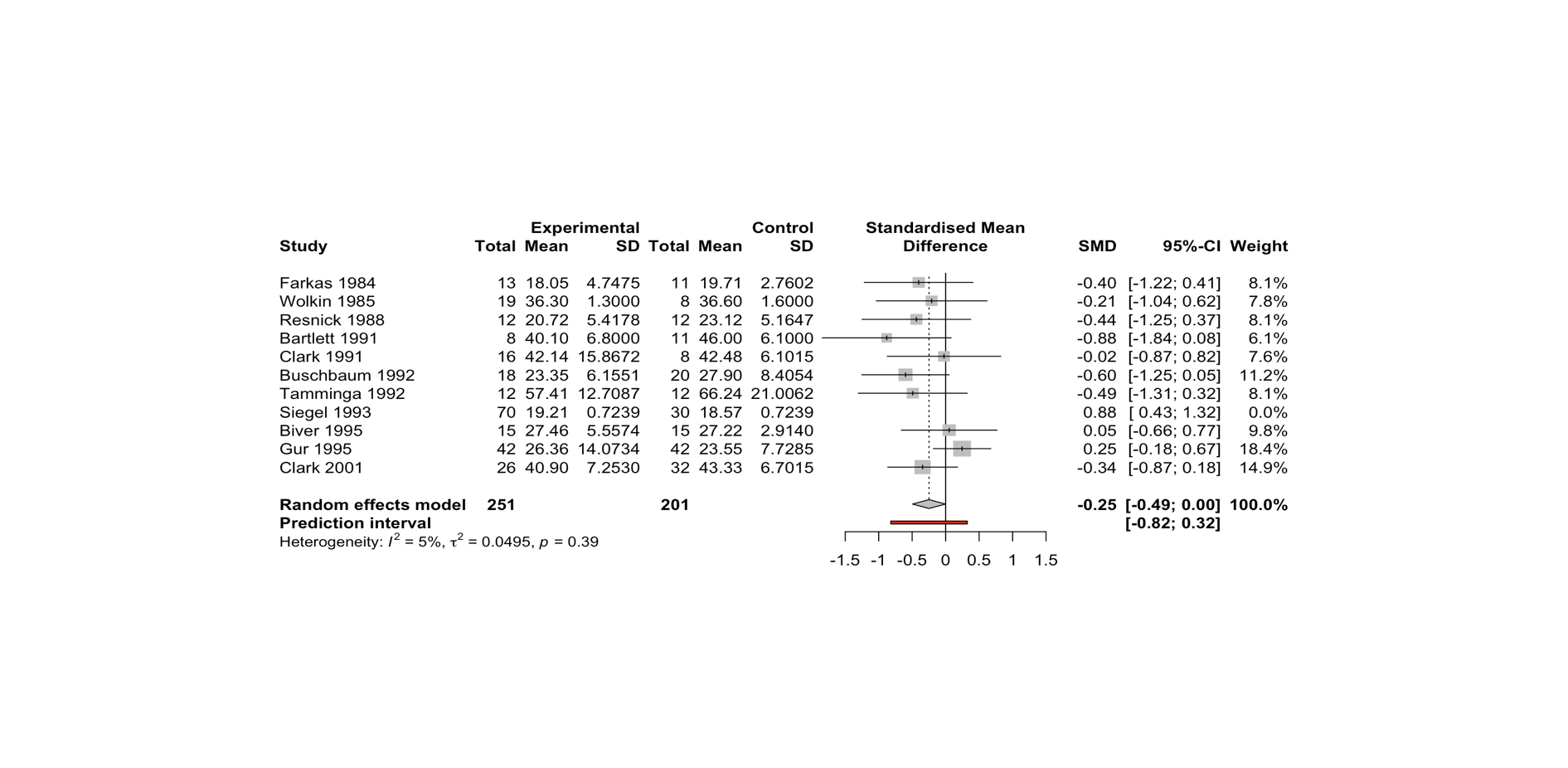


**Supplementary Fig 6.2 Meta-analysis of studies reporting basal ganglia absolute CMRGlu with outlier study (Siegel 1993) removed:**

**Forest plot showing results of random-effects meta-analysis**

**Supplementary Fig 8 Meta-analysis of studies reporting temporal absolute CMRGlu:**

**Forest plot showing results of random-effects meta-analysis**

**Supplementary Fig 9 Meta-analysis of studies reporting temporal normalised CMRGlu:**

**Forest plot showing results of random-effects meta-analysis**


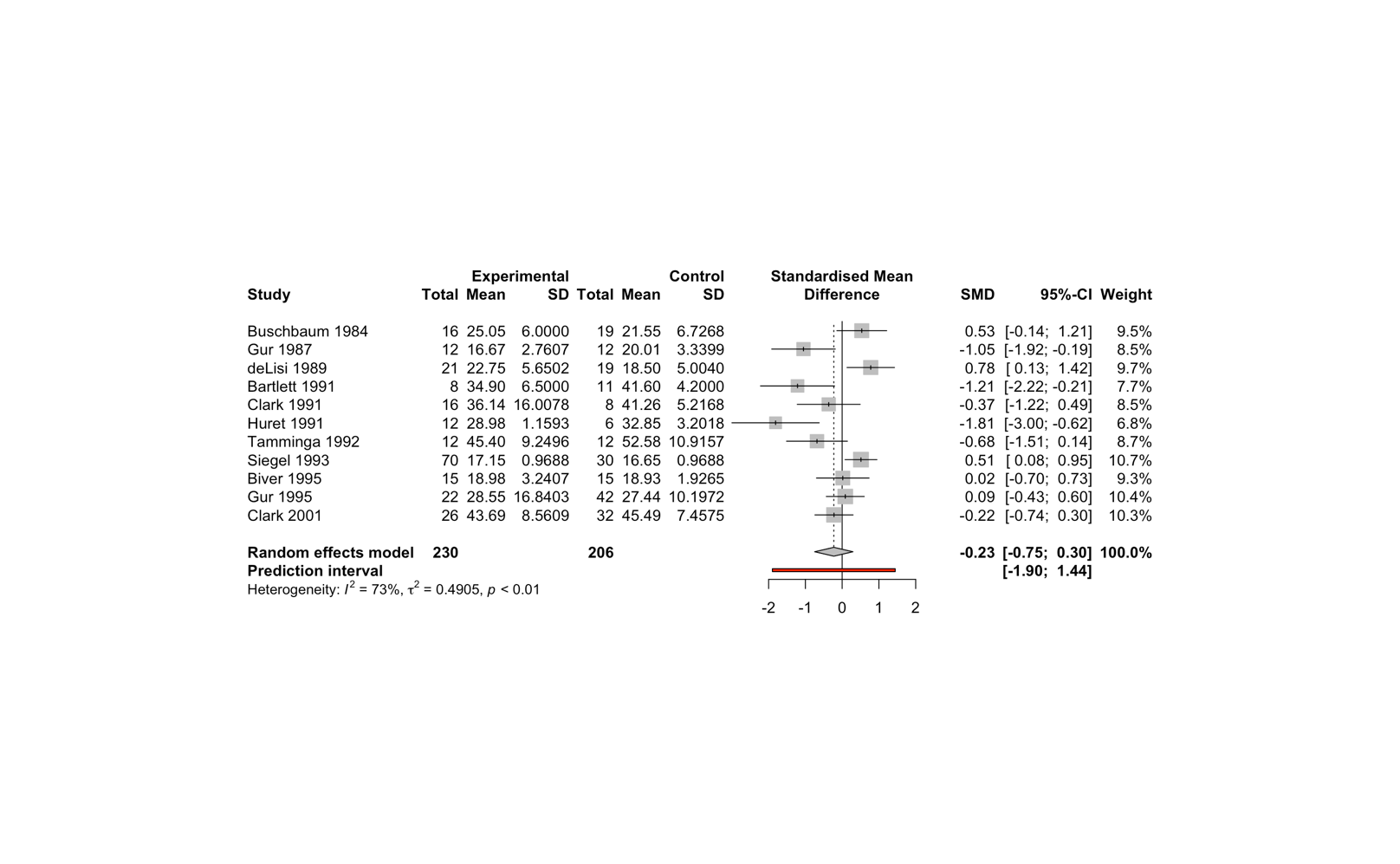

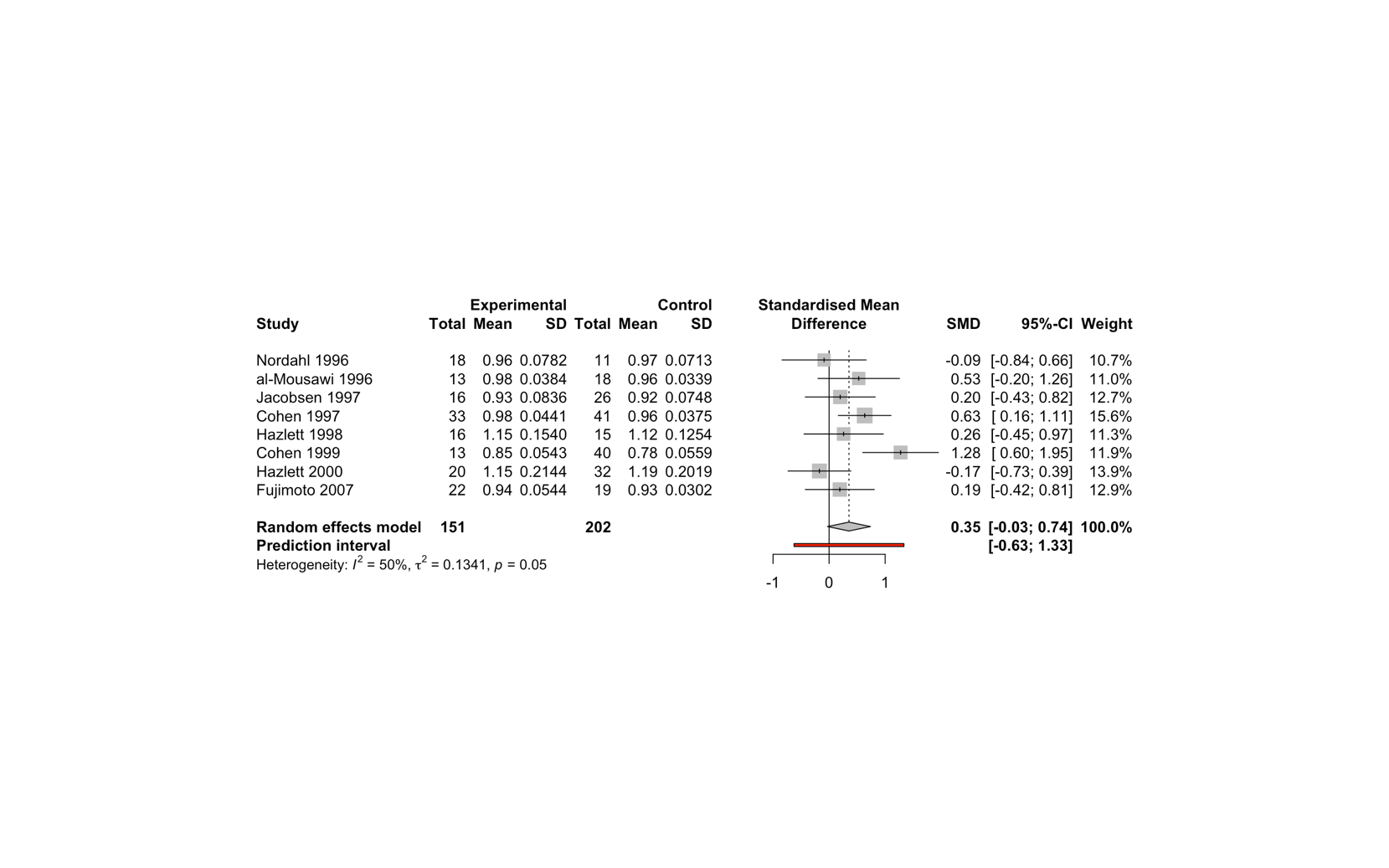


**Supplementary Fig 10 Meta-analysis of studies reporting parietal absolute CMRGlu:**

**Forest plot showing results of random-effects meta-analysis**

**Supplementary Fig 11 Meta-analysis of studies reporting parietal normalised CMRGlu:**

**Forest plot showing results of random-effects meta-analysis**


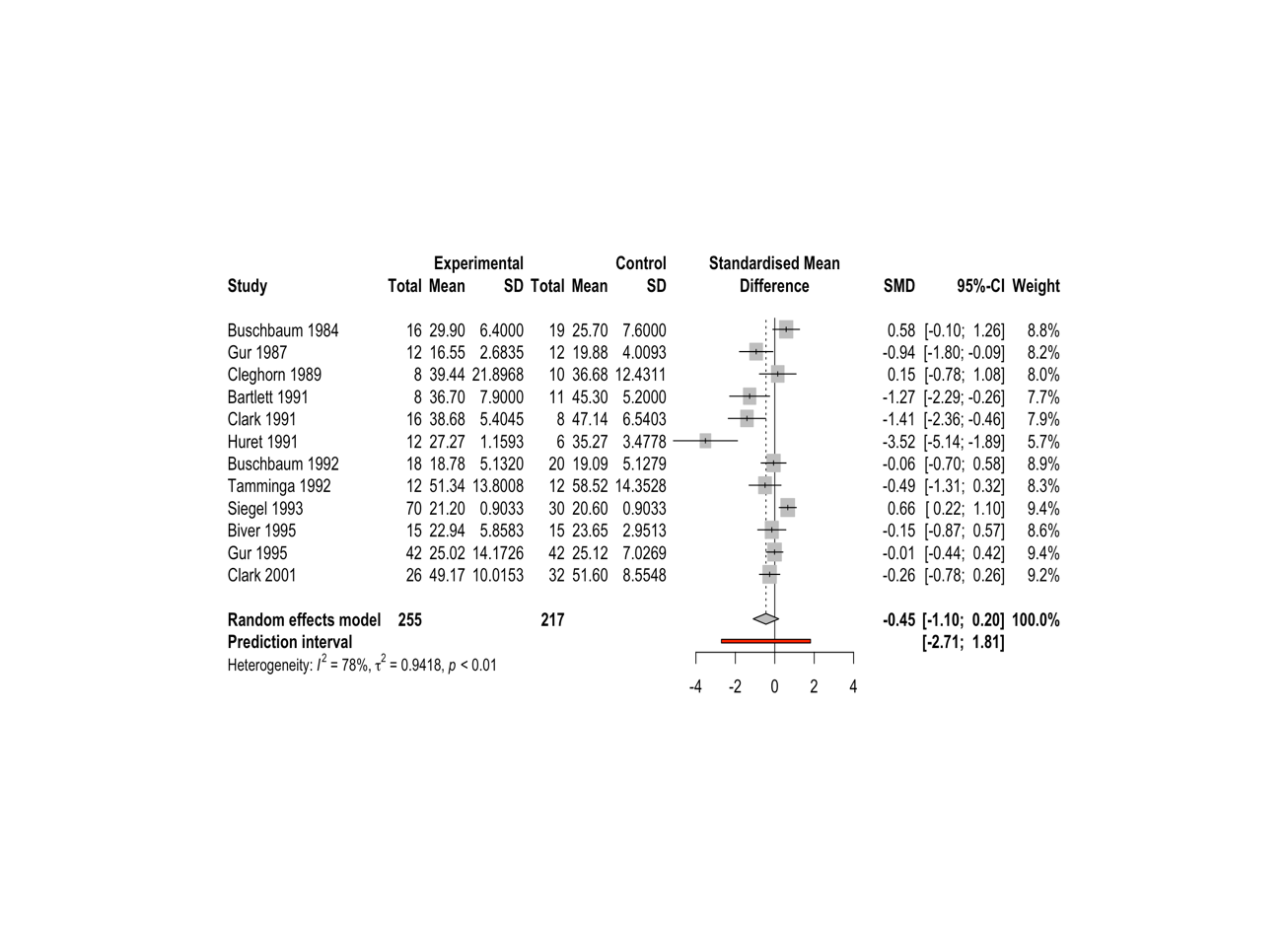

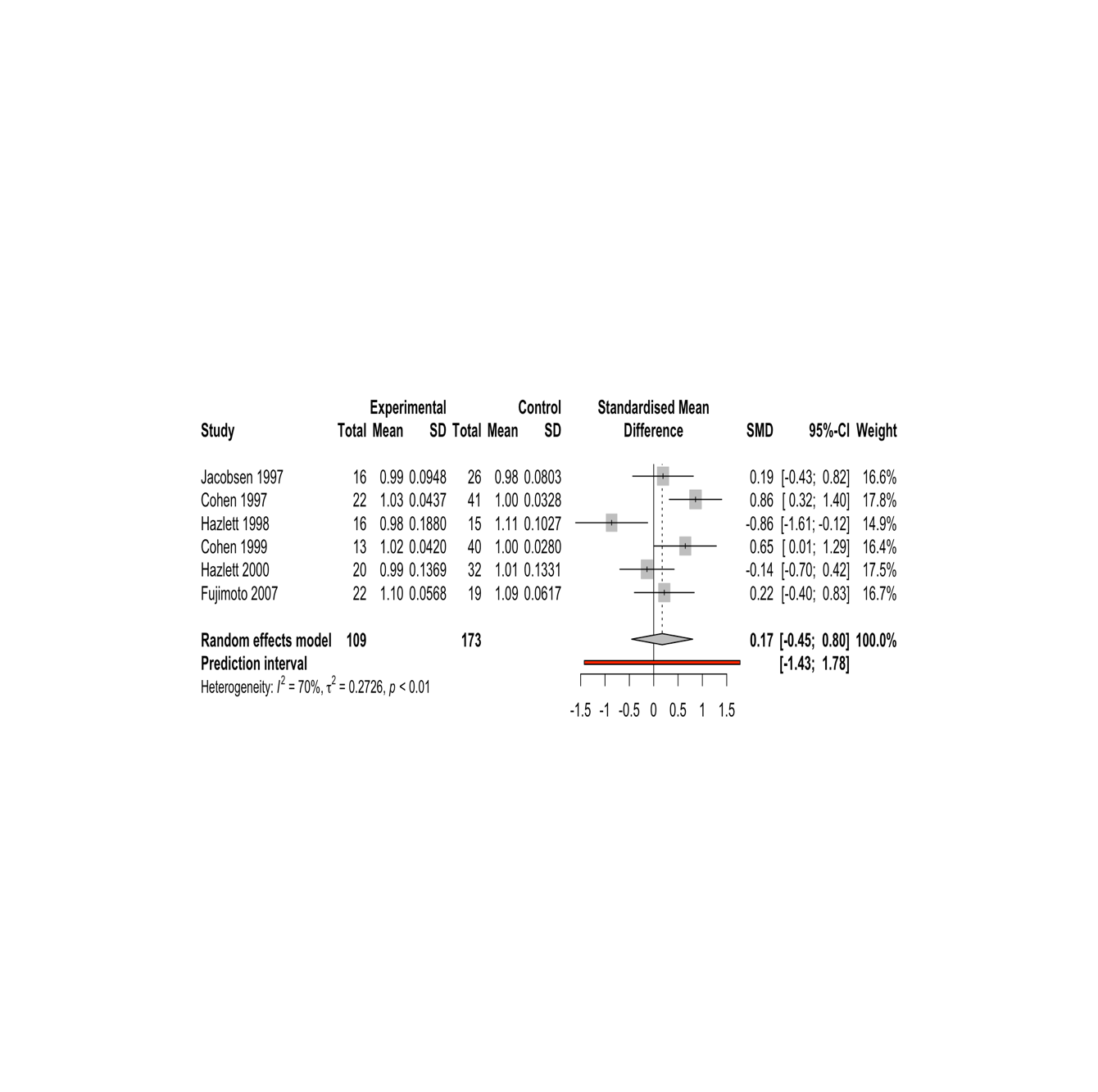


**Supplementary Fig 12 Meta-analysis of studies reporting occipital absolute CMRGlu:**

**Forest plot showing results of random-effects meta-analysis**


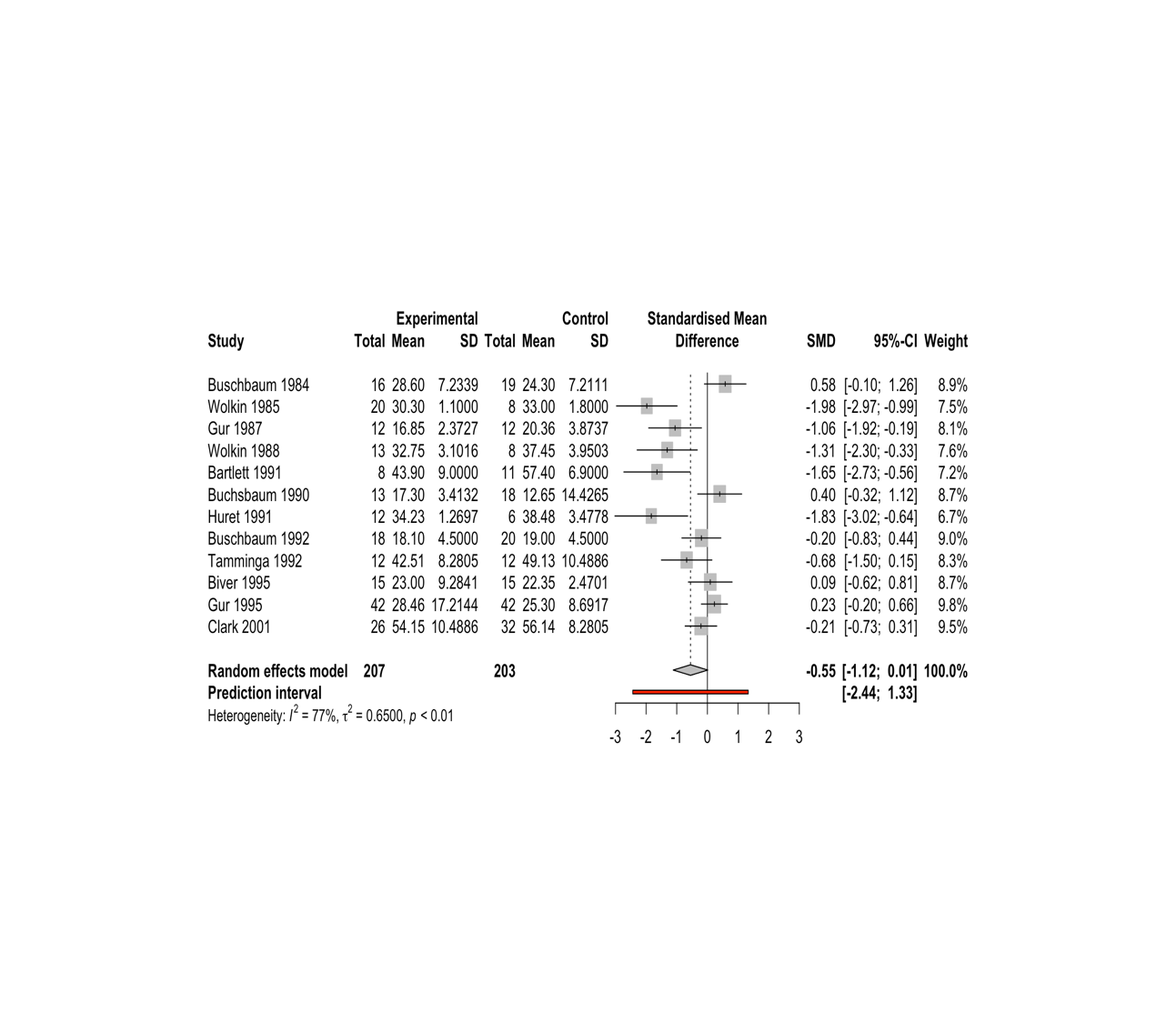

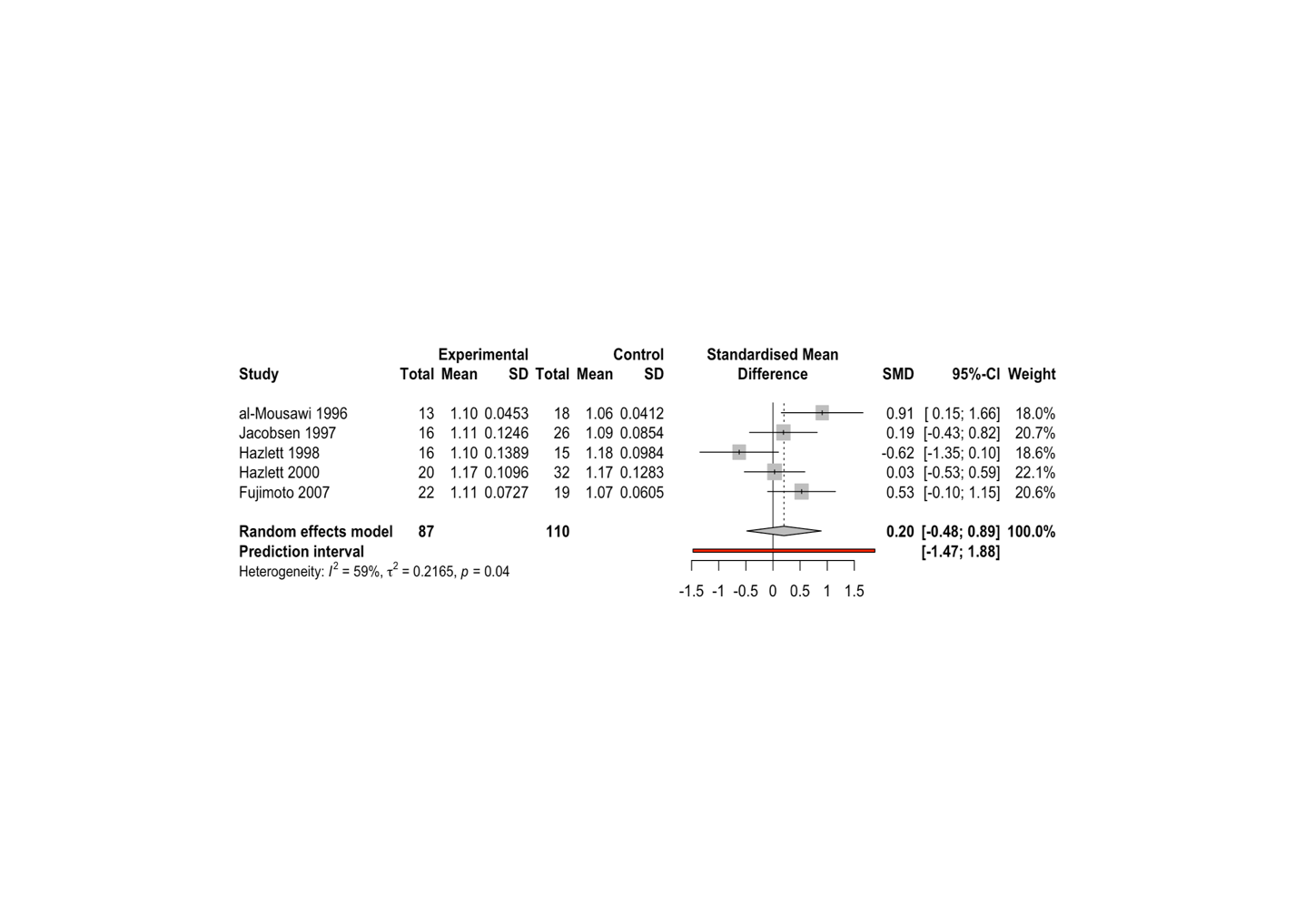


**Supplementary Fig 13 Meta-analysis of studies reporting occipital normalised CMRGlu:**

**Forest plot showing results of random-effects meta-analysis**

**Supplementary Fig 14 Meta-analysis of studies reporting thalamic absolute CMRGlu:**

**Forest plot showing results of random-effects meta-analysis**

**Supplementary Fig 15 Meta-analysis of studies reporting thalamic normalised CMRGlu:**

**Forest plot showing results of random-effects meta-analysis**


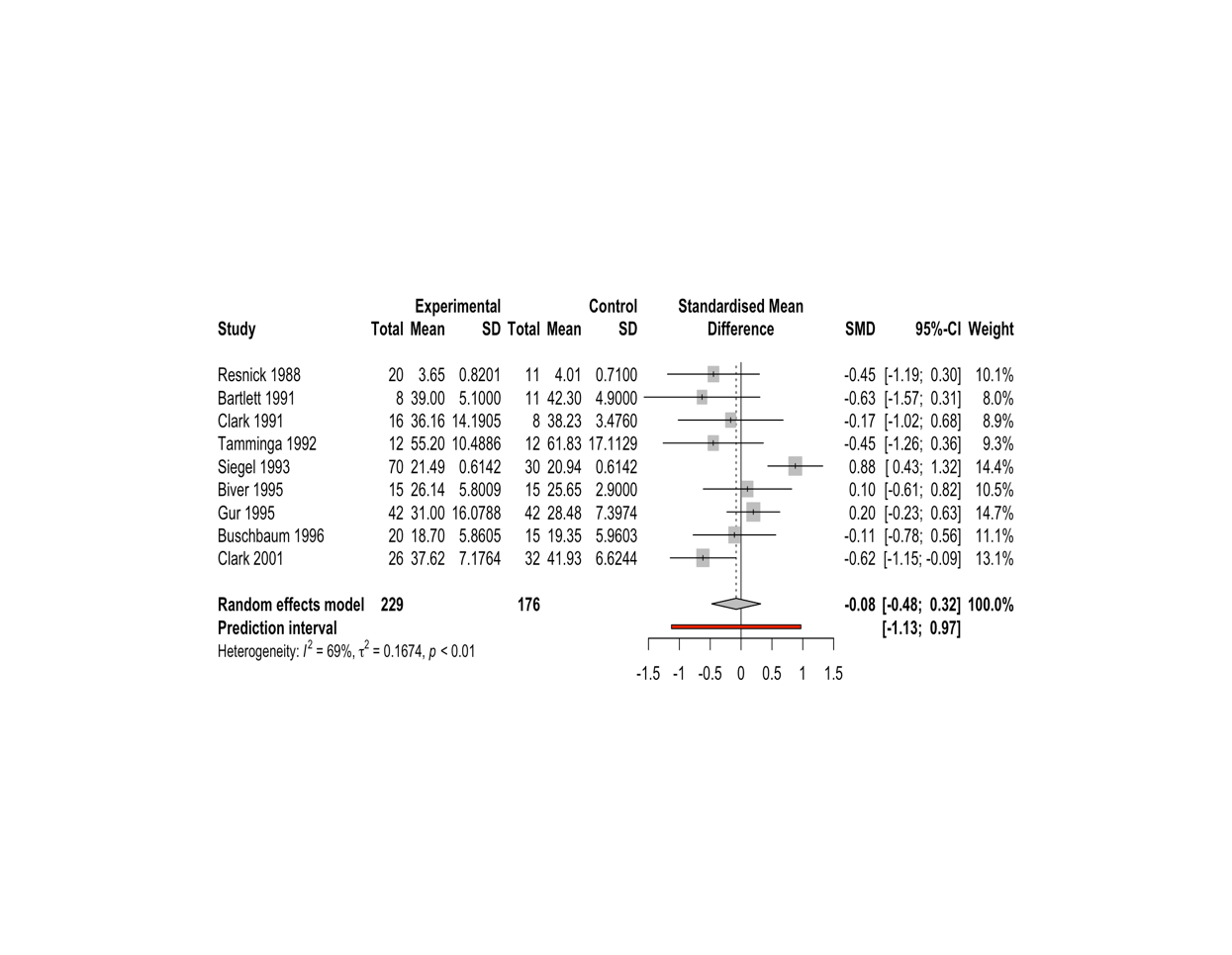

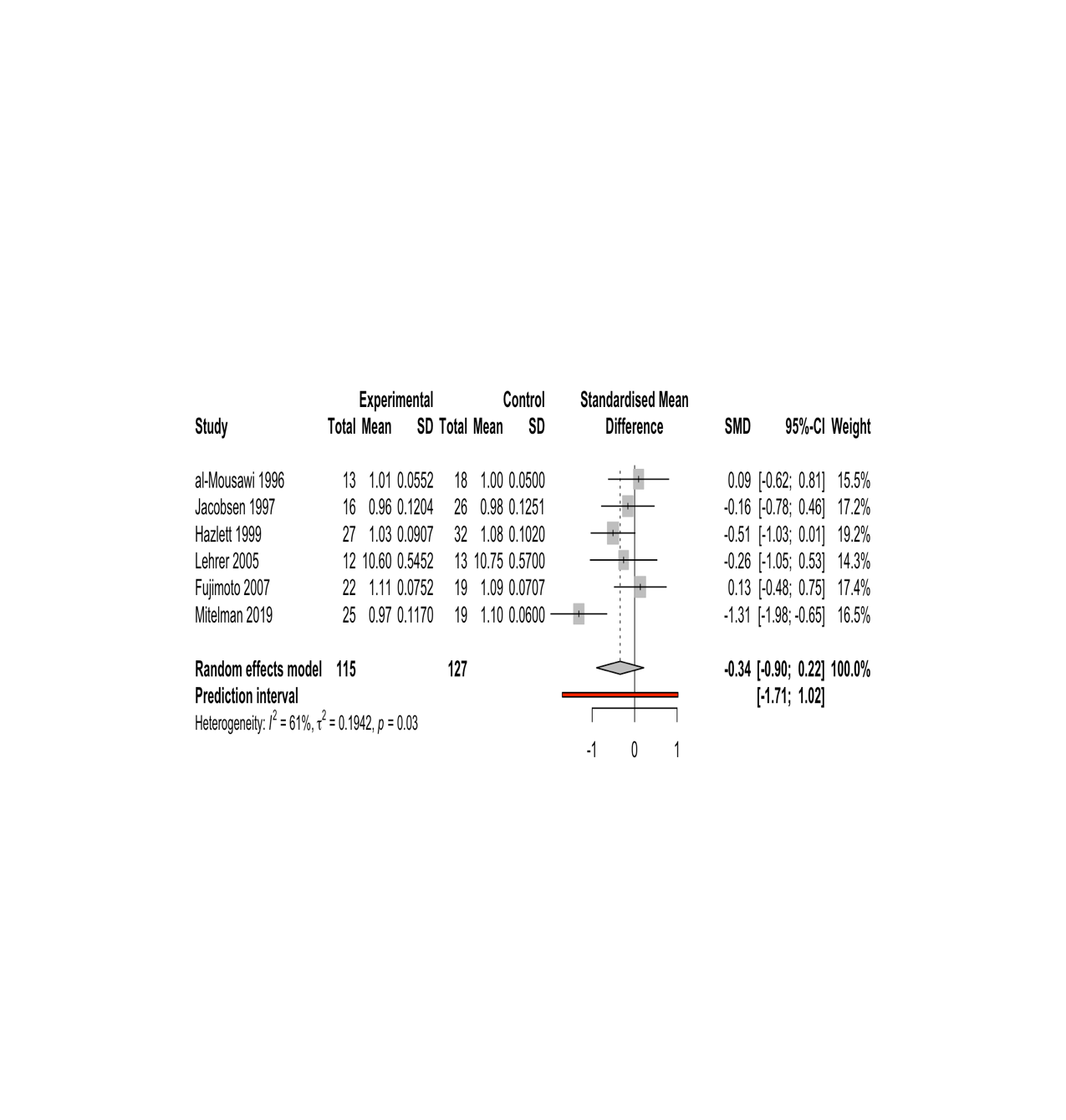

Supplement: Supplement [file EMS155472-supplement-Supplement.docx]
